# Hypoxic mesenchymal stem cells ameliorate acute kidney ischemia-reperfusion injury via enhancing renal tubular autophagy

**DOI:** 10.1186/s13287-021-02374-x

**Published:** 2021-06-28

**Authors:** Wei-Cheng Tseng, Pei-Ying Lee, Ming-Tsun Tsai, Fu-Pang Chang, Nien-Jung Chen, Chiang-Ting Chien, Shih-Chieh Hung, Der-Cherng Tarng

**Affiliations:** 1 Division of Nephrology, Department of Medicine, Taipei Veterans General Hospital, 201, Section 2, Shih-Pai Road, Taipei, 11217 Taiwan; 2 Faculty of Medicine, School of Medicine, National Yang-Ming University, Taipei, Taiwan; 3 Institute of Clinical Medicine, School of Medicine, National Yang-Ming University, Taipei, Taiwan; 4 Center for Intelligent Drug Systems and Smart Bio-devices (IDS2B), National Chiao-Tung University, Hsinchu, Taiwan; 5 Institute of Clinical Medicine, School of Medicine, National Yang Ming Chiao Tung University, Taipei, Taiwan; 6 Holistic Education Center, Tzu Chi University of Science and Technology, Hualien, Taiwan; 7 Department of Pathology and Laboratory Medicine, Taipei Veterans General Hospital, Taipei, Taiwan; 8 Institute of Microbiology and Immunology, School of Medicine, National Yang-Ming University, Taipei, Taiwan; 9 Department of Life Science, School of Life Science, National Taiwan Normal University, Taipei, Taiwan; 10 Integrative Stem Cell Center, Department of Orthopedics, and Institute of New Drug Development, New Drug Development Center, China Medical University, Taichung, Taiwan; 11 Institute of Biomedical Sciences, Academia Sinica, 128, Section 2, Academia Road, Taipei, 11529 Taiwan; 12 Department and Institute of Physiology, School of Medicine, National Yang-Ming University, Taipei, Taiwan; 13 Department of Biological Science and Technology, College of Biological Science and Technology, National Chiao-Tung University, Hsinchu, Taiwan

**Keywords:** Acute kidney injury, Autophagy, Hypoxic mesenchymal stem cells, Ischemia-reperfusion injury

## Abstract

**Background:**

Acute kidney injury (AKI) is an emerging global healthcare issue without effective therapy yet. Autophagy recycles damaged organelles and helps maintain tissue homeostasis in acute renal ischemia-reperfusion (I/R) injury. Hypoxic mesenchymal stem cells (HMSCs) represent an innovative cell-based therapy in AKI. Moreover, the conditioned medium of HMSCs (HMSC-CM) rich in beneficial trophic factors may serve as a cell-free alternative therapy. Nonetheless, whether HMSCs or HMSC-CM mitigate renal I/R injury via modulating tubular autophagy remains unclear.

**Methods:**

Renal I/R injury was induced by clamping of the left renal artery with right nephrectomy in male Sprague-Dawley rats. The rats were injected with either PBS, HMSCs, or HMSC-CM immediately after the surgery and sacrificed 48 h later. Renal tubular NRK-52E cells subjected to hypoxia-reoxygenation (H/R) injury were co-cultured with HMSCs or treated with HMSC-CM to assess the regulatory effects of HSMCs on tubular autophagy and apoptosis. The association of tubular autophagy gene expression and renal recovery was also investigated in patients with ischemic AKI.

**Result:**

HMSCs had a superior anti-oxidative effect in I/R-injured rat kidneys as compared to normoxia-cultured mesenchymal stem cells. HMSCs further attenuated renal macrophage infiltration and inflammation, reduced tubular apoptosis, enhanced tubular proliferation, and improved kidney function decline in rats with renal I/R injury. Moreover, HMSCs suppressed superoxide formation, reduced DNA damage and lipid peroxidation, and increased anti-oxidants expression in renal tubular epithelial cells during I/R injury. Co-culture of HMSCs with H/R-injured NRK-52E cells also lessened tubular cell death. Mechanistically, HMSCs downregulated the expression of pro-inflammatory interleukin-1β, proapoptotic Bax, and caspase 3. Notably, HMSCs also upregulated the expression of autophagy-related LC3B, Atg5 and Beclin 1 in renal tubular cells both in vivo and in vitro. Addition of 3-methyladenine suppressed the activity of autophagy and abrogated the renoprotective effects of HMSCs. The renoprotective effect of tubular autophagy was further validated in patients with ischemic AKI. AKI patients with higher renal LC3B expression were associated with better renal recovery.

**Conclusion:**

The present study describes that the enhancing effect of MSCs, and especially of HMCSs, on tissue autophagy can be applied to suppress renal tubular apoptosis and attenuate renal impairment during renal I/R injury in the rat. Our findings provide further mechanistic support to HMSCs therapy and its investigation in clinical trials of ischemic AKI.

**Supplementary Information:**

The online version contains supplementary material available at 10.1186/s13287-021-02374-x.

## Introduction

Acute kidney injury (AKI), namely an abrupt decrease in kidney function, is a frequent clinical syndrome with incidence rates of approximately 7–18% of all hospital inpatients and 20–200 per million population in the community [1]. AKI is associated with increased medical expenditure, higher morbidity, and considerable mortality [2, 3]. Renal ischemia-reperfusion (I/R) injury is a common cause of AKI and occurs in various clinical settings including shock, major surgeries, trauma, and sepsis [4, 5]. The interruption of renal blood flow (ischemia) and subsequent reperfusion result in a mismatch of local tissue oxygen supply and demand as well as imbalance of nutrient delivery and waste removal in the cells of the kidney [5]. This imbalance initiates renal cell death, oxidative stress, inflammation, and subsequent organ dysfunction [5, 6]. The extensive molecular degeneration caused by oxidative stress further raises the levels of DNA oxidation and lipid peroxidation byproducts which are implicated in the progression of acute renal cell injury [7]. The proximal tubule is the main injured site during ischemia in mammalian kidneys, and the death of injured proximal tubular epithelial cells constitutes the primary feature of renal I/R injury and the subsequent loss of renal function [8]. During the I/R injury, damaged renal tubular epithelial cells also generate pro-inflammatory and chemotactic cytokines, thereby further aggravating the extent of renal inflammation and injury [5, 6, 8]. Although great efforts have been made to improve the treatment of AKI, there are still no proven effective therapeutic agents found to be protective in AKI [3]. Therefore, to develop an innovative therapeutic approach for ischemic AKI remains an unmet medical need.

Autophagy is a highly conserved lysosomal degradation and recycling process of damaged or superfluous organelles and macromolecules [9]. Autophagy prevents cell damage and promotes cell survival in response to energy shortage and cytotoxic insults [9]. Dysregulated autophagy contributes to the pathogenesis of various human pathologies, ranging from cancer, neurodegenerative disorders to kidney diseases [9, 10]. Recently, autophagy has been implicated in the pathophysiology of AKI [10]. Proximal tubule-specific deletion of autophagy-related gene 5 (*Atg5*) in mice increases the susceptibility to I/R-induced renal injury, and worsens tubular apoptosis and renal impairment [11]. Mice with targeted deletion of another autophagy associated gene *Atg7* are also more sensitive to renal I/R injury than their wild-type littermates [12]. Collectively, autophagy plays a renoprotective role during renal I/R injury and strategies to enhance the autophagy may be a novel therapeutic approach to treat AKI.

Mesenchymal stem cells (MSCs) represent a new frontier of therapeutic strategies to repair various kinds of kidney injury for their anti-oxidative, anti-inflammatory and tissue repair properties [13–18]. The therapeutic effects of the MSCs can be mediated by direct “homing” to the injured organs and transdifferentiation into injured cells or by secretion of beneficial factors that exert an indirect paracrine/endocrine effect on damaged cells and tissue [16, 17]. Although MSCs have demonstrated a renoprotective effect in AKI, detailed mechanism by which MSCs lessen renal I/R injury is still incompletely understood [16, 17]. Many challenges still exist while applying the MSCs into clinical AKI patients because the efficacy of MSCs is influenced by several important aspects including administration routes and culture conditions [19–21]. Direct intra-arterial delivery that bypasses the lung can increase the number of accumulated MSCs in the I/R-injured kidneys [22], but whether this increased retention of MSCs confers a better renoprotection remains unclear. Furthermore, MSCs lose the proliferative and trophic potency over time during culture [23]. Preconditioning of the MSCs by hypoxia before the therapeutic application increases their secretion of beneficial trophic factors and augments the anti-oxidative property [20, 24–27]. Our previous works have found that hypoxic mesenchymal stem cells (HMSCs) can circumvent replicative senescence, enhance the proliferation rate and differentiation potential, and secrete more anti-inflammatory cytokines in vitro [28, 29]. Moreover, the hypoxic culture also increases the engraftment of MSCs, enhances angiogenesis, and prevents limb amputation in mice with hindlimb ischemia as compared to normoxic MSCs [30], suggesting that the hypoxia-preconditioning may be also a promising approach for better therapeutic benefits of MSCs in ischemic AKI. Recently, MSCs are reported to enhance the autophagy, promote β-amyloid clearance and increase the viability of co-cultured β-amyloid-treated neuronal cells [31]. Moreover, administration of MSCs also increases the autophagy level and decrease pulmonary cell death in mice with acute lung injury and co-cultured pulmonary micro-vascular endothelial cells [32]. Till now, whether MSCs mitigate renal I/R injury via modulating tubular autophagy has not yet been elucidated.

The present study aimed to investigate whether hypoxic culture, homing/paracrine actions, and different administration routes influence the renoprotective efficacy of MSCs in ischemic AKI. We found that HMSCs had a superior anti-oxidative effect than normoxia-cultured MSCs in renal I/R injury. We further demonstrated that intra-renal arterial administration of HMSCs attenuated inflammation and tubular cell apoptosis and kidney function decline in renal I/R injury through upregulation of renal tubular autophagy. The protective effect of tubular autophagy in I/R injury was further confirmed in patients with AKI. This observation provides mechanistic support of HMSCs therapy in renal I/R injury through modulating tubular autophagy and helps apply HMSCs in treating AKI patients.

## Materials and methods

### Isolation and culture of HMSCs

MSCs were isolated from 7-week-old male Sprague-Dawley rats as described previously [33]. Briefly, the ends of each tibia and femur were removed to expose the marrow. Thereafter, the bones were inserted into adapted centrifuge tubes and centrifuged at 400 *g* for 1 min to collect the marrow. The cell pellet of the marrow was then resuspended in 3 mL of complete medium [α-MEM (α-minimal essential medium; Gibco, Gaithersburg, MD), supplemented with 16.6% fetal bovine serum (FBS, ThermoFisher, Waltham, MA), 100 units/mL penicillin, 100 μg/mL streptomycin, and 2 mM L-glutamine], filtered through a 70-μm nylon mesh filter, and seeded in dishes. After 24 h, non-adherent cells were washed away with phosphate-buffered saline (PBS), and 10 mL of fresh complete medium was added. After reaching subconfluence around 2 weeks later, adherent cells were washed with PBS and detached by incubation in 4 mL of 0.25% trypsin/1 mM ethylenediaminetetraacetic acid (Invitrogen, Carlsbad, CA) at 37 °C for 2 min. The cells were then expanded by plating at 100 cells/cm^2^ and grown in the complete medium which was refreshed twice per week.

The normoxia-cultured MSCs were expanded at 37 °C in a humidified incubator (model 3130; ThermoFisher) containing 74% N_2_, 5% CO_2_, and 21% O_2_. For hypoxic culture, HMSCs were cultured in a gas mixture composed of 94% N_2_, 5% CO_2_, and 1% O_2_. The hypoxic gas mixture was maintained by a compact gas oxygen controller (ProOx P110, BioSpherix, Lacona, NY) supplied with N_2_ gas. If the O_2_ concentration rose above the desired level, N_2_ gas was automatically delivered into the system to displace the excess O_2_.

For collection of condition medium (CM), HMSCs were plated at 5000 cells/cm^2^ and incubated in the complete medium for one day. Afterwards, the cells were washed three times with PBS, and the complete medium was replaced with serum-free α-MEM to generate HMSC-CM. The HMSC-CM was collected after 48 h of culture, centrifuged at 2000 rpm for 10 min, and passed through a 0.2-μm filter. The HMSC-CM was further concentrated using centrifugal filter units with 5 kDa cut-off (Millipore, Billerica, MA).

### Characterization of HMSCs

To analyze the expression levels of stem cell surface marker proteins, HMSCs were harvested by TryPLE Select (ThermoFisher). Afterwards, 1 × 10^6^ cells were incubated with either fluorescein isothiocyanate (FITC)- or phycoerythrin-conjugated antibodies against mouse CD11b (BioLegend, San Diego, CA), CD29 (BioLegend), CD44 (BioLegend), CD45 (BioLegend), CD73 (Bioss, Woburn, MA), CD90 (BioLegend) and CD105 (Bioss). To analyze CD31 expression, the HMSCs were stained with rabbit anti-mouse anti-CD31 antibody (Santa Cruz Biotechnology, Santa Cruz, CA) followed by FITC-conjugated goat anti-rabbit secondary antibody (Jackson ImmunoResearch, West Grove, PA). Ten thousand labeled cells were acquired and analyzed using a FACS Canto II flow cytometer (BD Biosciences, San Diego, CA).

Differentiation of MSCs was induced as previously described with slight modification [33]. For in vitro differentiation into osteoblasts and adipocytes, cells were seeded at a density of 1 × 10^4^/cm^2^ and induced in osteoblast induction medium [α-MEM supplemented with 10% FBS, 10^−8^ M dexamethasone (Sigma-Aldrich, St Louis, MO), 10 mM β-glycerol phosphate (Sigma-Aldrich) and 50 μM ascorbate-2-phosphate (Sigma-Aldrich)] and adipocyte induction medium [α-MEM supplemented with 10% FBS, 10^−7^ M dexamethasone, 50 μM indomethacin (Sigma-Aldrich), 0.45 mM 3-isobutyl-1-methyl-xanthine (Sigma-Aldrich) and 10 μg/mL insulin (Sigma-Aldrich)] for 2 weeks. For in vitro differentiation into chondrocytes, 5 × 10^7^ cells were centrifuged (300×*g*, 4 °C) to form a high-density micromass culture pellet. The pellet was then continuously cultured in chondrocyte induction medium [serum-free α-MEM supplemented with 10^−7^ M dexamethasone, 1% (vol/vol) insulin-transferrin-selenium premix (Corning Inc., Corning, NY), 50 μM ascorbate-2-phosphate, 40 μg/mL (wt/vol) proline (Sigma-Aldrich), and 10 ng/mL (wt/vol) transforming growth factor-β1 (R&D systems, Minneapolis, MN)] for 3 weeks. After the appearance of morphological features of differentiation, cells treated in the induction medium of osteoblasts and adipocytes were stained with alizarin red S (Sigma-Aldrich) and oil red O (Sigma-Aldrich), respectively. The cell pellet cultured in the chondrocyte induction medium was prepared in paraffin sections, stained with Alcian blue (Millipore), and counterstained with nuclear fast red (Sigma-Aldrich).

### Animal model of renal I/R injury

Adult Sprague-Dawley rats (male, 8 weeks old, approximately 300 g) were purchased from the Laboratory Animal Center of the National Yang-Ming University (Taipei, Taiwan) and raised in a sound-attenuated, temperature-controlled (22 ± 1 °C) room with a 12-h light/dark cycle. Standard rodent chow and drinking water were supplied ad libitum. The renal I/R injury model was performed as previously described [34, 35]. Briefly, for induction of total ischemia in the kidney, the left renal artery was clamped with a nontraumatic vascular clamp for 45 min. Afterward, reperfusion was initiated by releasing the clamp, and the right kidney was removed simultaneously. The intraoperative body temperature, cardiac function, and respiratory rates and pattern of the rats were monitored closely. The respiratory rates and pattern were assessed by visual observation. The cardiac function was assessed by monitoring heart rate, mucous membrane color, and capillary refill time. The core temperature was measured by a rectal thermometer. During the surgery, the rats were kept on a heating pad to maintain the core temperature between 36.5 and 37 °C. After surgery, either PBS, MSCs (5 × 10^5^ cells) or HMSCs (5 × 10^5^ cells) were administered via intra-renal arterial (IA) or intraperitoneal routes (IP). To investigate the paracrine effect, concentrated HMSC-CM was injected intraperitoneally. Sham-operated animals underwent similar operative procedures without receiving clamping of the left renal artery and right nephrectomy. The rats were humanely euthanized by decapitation under anesthesia 48 h later to collect the blood and kidney tissue samples for further analyses. Serum levels of blood urea nitrogen (BUN) and creatinine were determined by an Olympus AU-2700 autoanalyzer (Olympus Ltd, Tokyo, Japan). Because estimating the effect size of stem cell therapy is difficult, the conventional sample size calculation cannot be applied. We instead utilized the “resource equation” method, which is commonly used in experimental animal studies [36]. All experimental procedures conformed to the Guide for the Care and Use of Laboratory Animals published by the National Institutes of Health. The study was approved by the Institutional Animal Care and Use Committee of the National Yang-Ming University and the Taipei Veterans General Hospital under the license numbers of 991261 and 2017-075, respectively.

### Tracking analyses of HMSCs in vivo

The HMSCs were labeled with a commercial PKH67 staining kit (PKH67GL-1KT, Sigma-Aldrich), according to the manufacturer’s instructions. Briefly, after washed by PBS, HMSCs were mixed with PKH67 solution and incubated for 5 min at room temperature. Unbound PKH67 molecules were quenched by adding 2 mL of 10% bovine serum albumin, and then the cell suspension was centrifuged. The supernatant was discarded and the PKH67-labeled HMSCs were resuspended in PBS for further use. Immediately after the renal I/R injury, the PKH67-labelded HMSCs were administered via either IA or IP route. The I/R-injured rats were sacrificed 48 h later and the kidneys were harvested. After embedded in the optimal cutting temperature compound, the kidney tissues were cut into 4-μm sections, fixed by 4% paraformaldehyde for 15 min at room temperature, and then counterstained with 4′, 6-diamidino-2-phenylindole (DAPI, Sigma-Aldrich). The emission of PKH67 was detected by using the standard filter setup for FITC.

### Histology analysis and immunohistochemical staining

The kidney tissue was fixed with 4% phosphate-buffered formalin, embedded in paraffin block, and cut into 4-μm sections. Sections were stained with periodic acid-Schiff (PAS) as previously described [34]. Tubular injury (denudation of tubular cells, loss of brush border, flattening of tubular cells, formation of intratubular casts) was scored from 0 to 4 (0, no changes; 1, changes affecting < 25%; 2, changes affecting 25 to 50%; 3, changes affecting 50 to 75%; 4, changes affecting 75 to 100% of the section). Twenty randomly selected non-overlapping high-power fields (× 40 objective) were scored for each rat and the average for each group were then analyzed [34]. All scoring was performed in a blinded fashion.

Immunohistochemical staining was performed on formalin-fixed paraffin-embedded sections of kidneys as previously [37]. Briefly, after deparaffinization by xylene and rehydration by graded alcohol, consecutive 4-μm sections of kidneys were subjected to heat antigen retrieval in a microwave oven (650 W, 12 min) in 10 mM sodium citrate buffer (pH 6.0). Afterwards, endogenous peroxidase activity was quenched by 3% hydrogen peroxide for 10 min. Thereafter, tissue sections were incubated with the primary antibodies at 4 °C overnight and then with secondary antibody (Envision^+^Dual Link System-HRP, Dako, Glostrup, Denmark) for 30 min at room temperature. Signals were developed with diaminobenzidine and counterstained with Gill’s hematoxylin. Twenty randomly selected non-overlapping high-power fields (× 40 objective) at the renal cortex were evaluated for each mouse. Analysis of the diaminobenzidine-positive area was carried out using Image J with the “Threshold Colour” plug-in (version 1.16, https://imagejdocu.tudor.lu/plugin/color/threshold_colour/start#threshold_colour) as previously reported [37]. Details of the antibodies used in the immunohistochemistry were listed in the Supplemental Table S1.

### Renal reactive oxygen species (ROS) detection

ROS formation in the I/R-injured kidneys was determined by in vivo detection of 2-methyl-6-(p-methoxyphenyl)-3,7-dihydroimidazo [1,2-alpha]pyrazin-3-one (MCLA)-enhanced chemiluminescence from the kidney surface and by ex vivo detection of lucigenin-enhanced chemiluminescence from the kidney homogenate as described previously with slight modifications [34, 38]. Both MCLA-enhanced and lucigenin-enhanced chemiluminescence are widely used as an indicator of superoxide production and have been utilized to estimate superoxide generation in tissue homogenates [38–40]. For in vivo detection of superoxide, the rats were anesthetized, kept on a heating pad to maintain the body temperature between 36.5 and 37 °C, and put in a dark box with a shielding plate. Only the renal window was left unshielded to detect the photons from the exposed kidney surface. MCLA (0.2 mg/mL/h, Cat. no. 87787, Sigma-Aldrich) was continuously infused via the right femoral vein and the PBS, MSCs, or HMSCs were administered via an intra-renal arterial catheter through the left femoral artery of I/R-injured rats. MCLA-enhanced chemiluminescence signals from the I/R-injured or sham-operated kidneys were measured continuously in a Chemiluminescence Analyzing System (CLD-110; Tohoku Electronic Industrial Co., Sendai, Japan). For ex vivo detection of renal ROS, the renal tissue samples were first homogenized with PBS (50 mg/mL) and put in a completely dark chamber of the Chemiluminescence Analyzing System (CLD-110). After a 60-s determination of background luminescence level, the superoxide levels in the kidney homogenates were measured by adding 0.5 mL of 0.1 mM lucigenin solution (pH 7.4, Sigma-Aldrich, M8010) to the kidney homogenate solution (0.2 mL). The lucigenin-enhanced chemiluminescence was then monitored continuously for an additional 540 s. The total amount of chemiluminescence was calculated by integrating of the area under the 540-s chemiluminescence curve after subtracting the background luminescence level. The amount of ROS was expressed as chemiluminescence counts/10 s.

### In situ detection of superoxide by dihydroethidium (DHE) staining

DHE staining was utilized to analyze the location of ROS production as described previously with modifications [34]. Briefly, 4-μm formalin-fixed paraffin-embedded sections of kidneys were deparaffinized, rehydrated, and subjected to heat antigen retrieval in a microwave oven (650 W, 12 min) in 10 mM sodium citrate buffer (pH 6.0). Endogenous peroxidase activity was quenched by 3% hydrogen peroxide for 10 min. Afterward, the section was incubated with mouse anti-rat CD68 antibody (GeneTex, Irvine, CA) at 4 °C overnight. Then, the section was incubated with FITC-conjugated secondary antibody (Jackson ImmunoResearch) at room temperature for 2 h. The section was stained with DHE (100 μM; Sigma-Aldrich) at room temperature for 15 min and counterstained with DAPI (Sigma-Aldrich).

### Terminal deoxynucleotidyl transferase-mediated digoxigenin-deoxyuridine triphosphate nick-end labeling (TUNEL) assay

To detect the apoptosis in the kidney, TUNEL assay was conducted using the Apoptag Peroxidase In Situ Apoptosis Detection kit (Millipore) according to the manufacturer’s instruction as previously described [34]. Apoptotic cells were visualized by diaminobenzidine (Dako). The presence of nuclear brown staining (TUNEL-positive) indicated apoptotic cells. Negative controls were stained with omission of the terminal deoxynucleotidyl transferase.

### Immunoblotting

Protein of kidney tissue and cells was extracted by the RIPA buffer containing 0.1% (v/v) protease inhibitor (Pierce) and the western blot analysis was performed as previously [37, 41]. Briefly, 50 μg of protein extract was loaded to 12% SDS-PAGE gels and transferred to polyvinylidene fluoride membranes. The membranes were incubated at 4 °C overnight with primary antibodies and then amplified by species-directed secondary antibodies. Signals were developed with a West Femto Chemiluminescent Substrate kit (Thermo Scientific, Hudson, NH). Bands were visualized and quantified using a ChemiDoc-It Imaging system (UVP, Cambridge, UK). Data were normalized to β-actin expression. Details of the antibodies used in the immunohistochemistry were listed in the Supplemental Table S1.

### Cell culture and in vitro hypoxia-reoxygenation model

Rat proximal renal tubular epithelial NRK-52E cells were obtained from the Food Industry Research and Development Institute (Hsinchu, Taiwan) and grown in Dulbecco’s modified Eagle’s medium (DMEM) containing 4 mM L-glutamine, 1.5 g/L sodium bicarbonate, 4.5 g/L glucose (Gibco), supplemented with 10% fetal bovine serum (ThermoFisher). The cells were incubated at 37 °C in a 5% CO_2_ atmosphere and passaged twice a week. In vitro hypoxia-reoxygenation model was performed as previously described [34]. Briefly, NRK-52E cells were plated in 35-mm dishes (Corning Inc.) at a density of 1 × 10^6^ cells/dish for one day. For hypoxia experiments, the cells were placed in a hypoxia C-chamber (BioSpherix) inside a standard CO_2_ incubator (model 3130; ThermoFisher) with a compact gas oxygen controller (ProOx P110; BioSpherix) to maintain oxygen concentration at 1% by introducing a gas mixture of 95% N_2_ and 5% CO_2_. After exposure to hypoxia for 24 h, the cells were returned to normoxic conditions (21% O_2_/74% N_2_/5% CO_2_) for reoxygenation for 6 h. Control cells were incubated in a regular cell culture incubator with 21% O_2_ constantly. At the end of the study, normoxia-cultured and hypoxia-reoxygenation (H/R)-injured NRK-52E cells were harvested with indicated buffers to collect cell lysates for biochemical analyses.

To investigate the effect of HMSCs on H/R, NRK-52E cells (5 × 10^5^) in a six-well plate were washed by PBS and then co-cultured with or without HMSCs (co-culture ratio 1:1) by Transwell (8 μm, Millipore) during H/R. For HMSC-CM treatment experiment, NRK-52E was treated with one-fold HMSC-CM after subjected to H/R. To suppress autophagy, 3-methyladenine (3-MA, 30 mM, Cayman Chemicals, Ann Arbor, MI) was added into the culture medium of NRK-52E cells in the indicated experiments.

### Cell viability assay

NRK-52E cells were seeded into 24-well plates and cell viability was measured by the colorimetric 3-[4,5-dimethylthiazol-2-yl]-2,5-diphenyltetrazolium bromide (MTT) assay as previously described [34]. After the NRK-52E cells were subjected to an H/R injury, 10 μl of MTT solution (5 mg/ml, Sigma-Aldrich) was added to each well and incubated for 1 h at 37 °C. Wells were emptied and blue formazan crystals were dissolved with 100 μl of MTT solubilization solution (10% Triton X-100, 0.1 N HCl in 2-propanol). The optical density was then analyzed at 570 nm using a TiterTek Multiskan microplate reader (Flow Laboratories, McLean, VA).

### Analysis of autophagy flux

Normoxia-cultured and H/R-injured NRK-52E cells were co-cultured with HMSCs in the presence or absence of 3-MA. Autophagic flux was measured by a Premo Autophagy Tandem Sensor RFP-GFP-LC3B kit (Life Technologies, Rockville, MD) according to the manufacturer’s instructions. Fluorescence images were acquired after 48 h of incubation and imaged using a ×63 objective on a confocal fluorescence microscope (FV10i, Olympus). The LC3B puncta of ten non-overlapping images were quantified using the Image J software.

### Analysis of autophagy-related gene expression in human samples

Human kidney specimens were obtained from diagnostic renal biopsies for patients with unexplained acute kidney injury in the Taipei Veterans General Hospital, Taiwan, from October, 2018, to October, 2019. Patients with pathological diagnosis of ischemic acute tubular injury were then enrolled in the study (n = 35). The renal biopsy specimen was microdissected and the glomeruli was removed. Subsequently, the total RNA from the kidney tissue was extracted by a NEBNext UltraRNA Library Prep Kit (New England Biolabs, Ipswich, MA). The RNA with a RIN ≥ 7.0 were used for further RNA sequencing. The cDNA libraries were constructed, pooled, and sequenced on an Illumina NovaSeq 6000 platform with 150 bp pair-ended reads. Quality control of the RNA-sequencing reads was through FastQC (version 0.11.8) and only reads with Phred quality scores ≥ 30 were retained. Thereafter, adaptors and lower-quality bases were trimmed by Trimmomatic (version 0.39) and the trimmed reads were then aligned to the human reference genome (GRCh38/hg38) with HISAT2 (version 2.2.1). The read counts of each sample were obtained using featureCounts (version 2.0.1). Autophagy-related gene expression was estimated as transcripts per million (TPM) [42, 43]. Baseline demographics, comorbidity, and laboratory data were recorded at the time of biopsy. Thereafter, the patients were followed periodically until February, 2021 to assess the recovery of renal function, which was defined as the occurrence of a glomerular filtration rate greater than or equal to the baseline value during the follow-up period. The institutional review board of the Taipei Veterans General Hospital approved the study under the license number 2018-06-008B and 2018-09-004C. All enrolled participants gave their informed consents.

### Statistical analysis

Categorical variables were expressed with numbers and percentages, and the numerical variables were shown as mean ± SEM for experimental data and as median plus interquartile range for clinical data. Between-group comparisons were determined by the Pearson’s chi-squared test, Fisher’s exact test, Student’s *t* test, Wilcoxon rank sum test, or one-way ANOVA with post hoc Tukey’s test where appropriate. Correlation between the autophagy-related gene and anti-oxidant genes was analyzed by the Pearson’s correlation analysis. A two-tailed *p* value < 0.05 was considered statistically significant. All analyses were performed with the R software (version 4.0.4, R Foundation for Statistical Computing, Vienna, Austria).

## Results

### Characterization of HMSCs

**Fig. 1:**
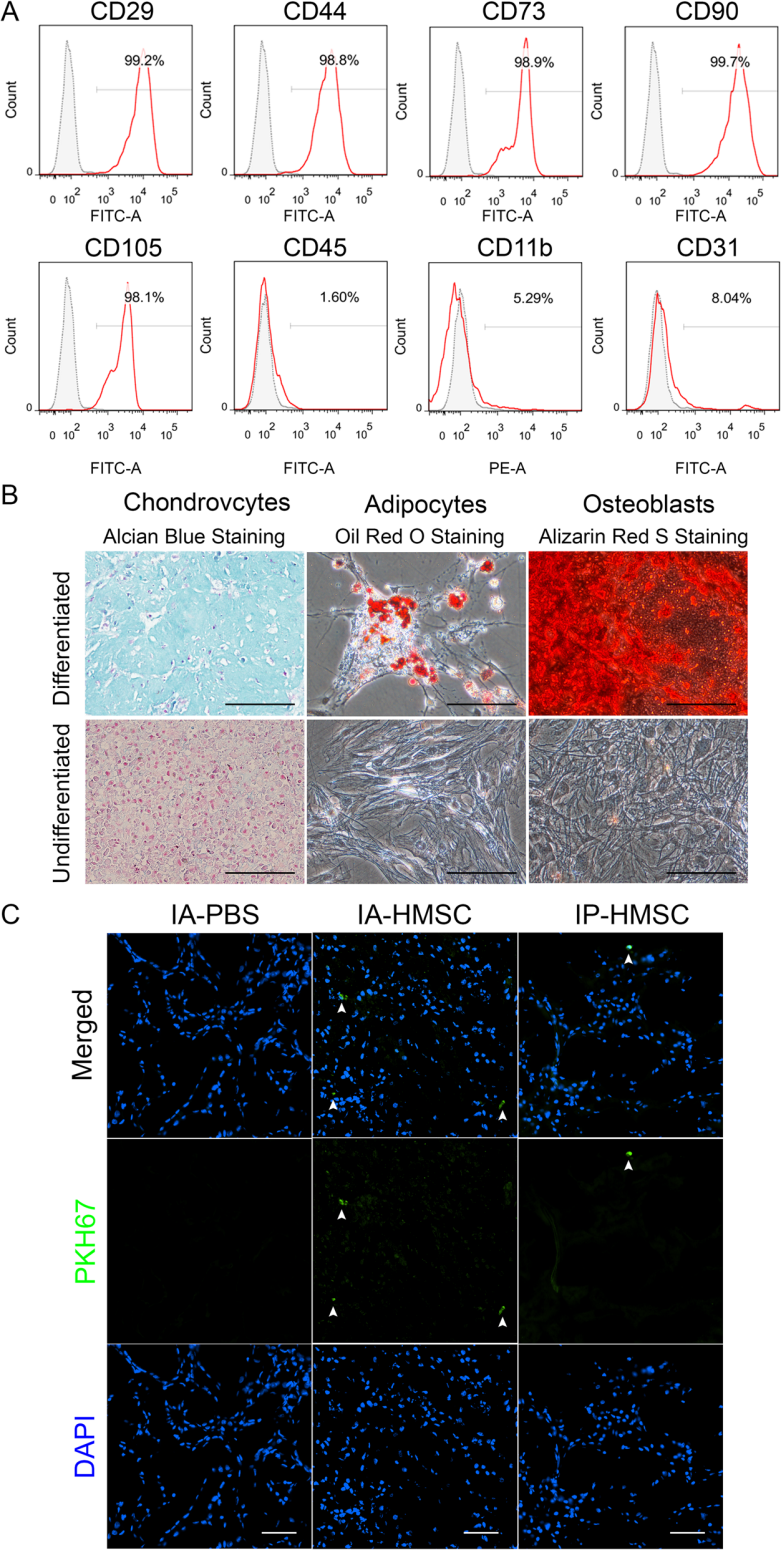
Characteristics and tracking of hypoxic rat mesenchymal stem cells (HMSCs) in ischemia-reperfusion injured rat kidneys. **a** HMSCs from the bone marrow of adult Sprague-Dawley rats were analyzed for the expression of surface markers (red lines) by flow cytometry. Gray shaded areas indicate unstained controls. **b** HMSCs had adipogenic, chondrogenic, and osteogenic differentiation abilities. Scale = 100 μm. **c** The rats with renal ischemia-reperfusion injury were administered with PKH67-labeled HMSCs or phosphate-buffered saline (PBS) via intra-renal arterial (IA) or intraperitoneal (IP) routes and sacrificed 48 h later. Fluorescence microscopy analysis revealed that PKH67-labeled HMSCs (arrowheads) retained the I/R-injured rat kidneys. *n* = 2 per group. Scale = 50 μm

To study the therapeutic roles of HMSCs on renal I/R injury, we first characterized the properties of the HMSCs. The HMSCs were isolated from the bone marrow of adult Sprague-Dawley rats, cultured at low density in a hypoxic atmosphere, and differentiated as previously described [33]. The rat HMSCs positively expressed stem cell markers including CD29, CD44, CD73, CD90, and CD105, but did not express hematopoietic cell markers, such as CD11b, CD31, and CD45 (Fig. 1a). The HMSCs were also able to differentiate into osteoblastic, chondrocytic, and adipocytic lineages (Fig. 1b).

### HMSCs confer a superior anti-oxidative effect in rats with I/R injury

**Fig. 2:**
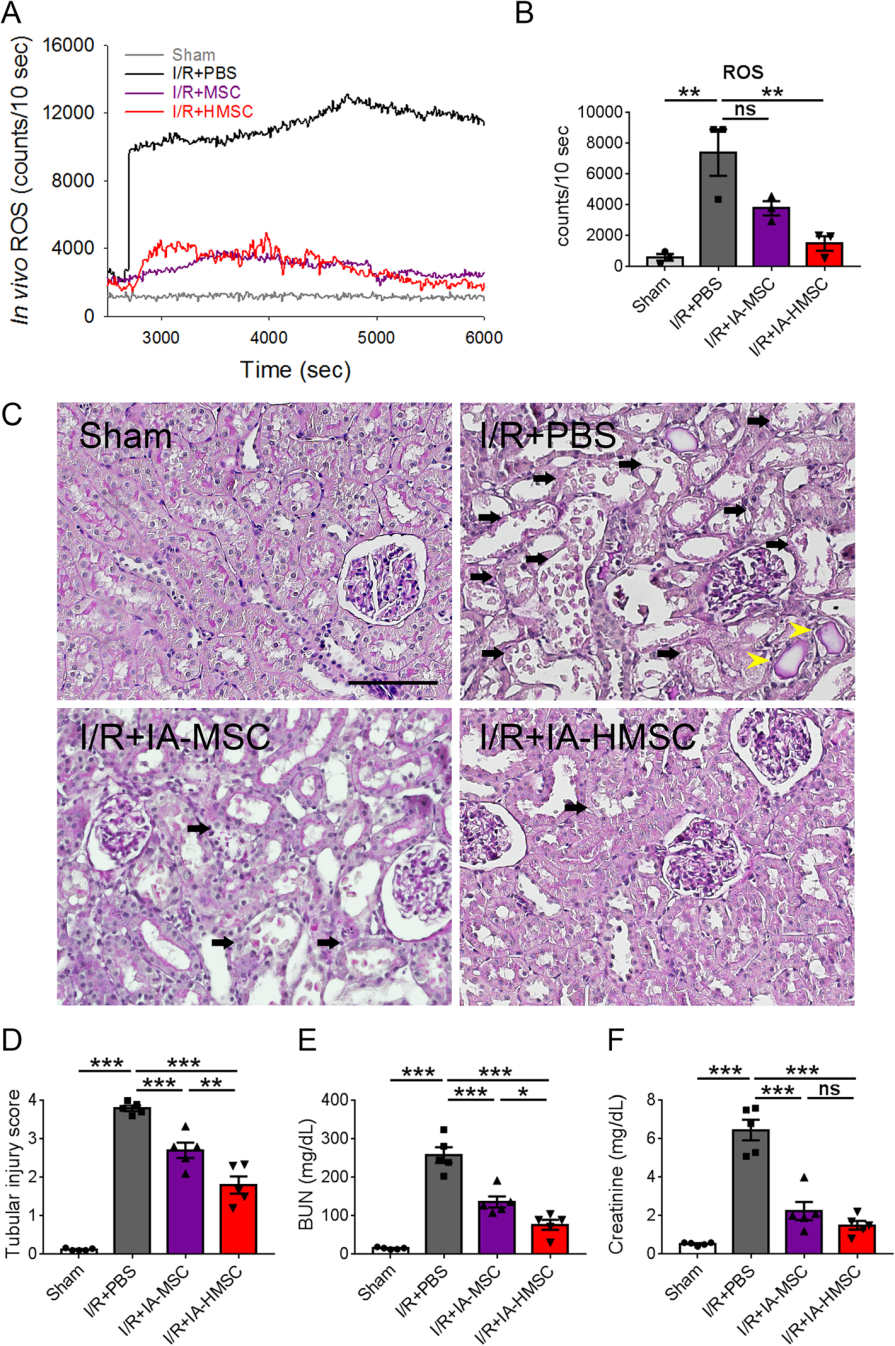
Effect of hypoxic rat mesenchymal stem cells (HMSCs) on oxidative stress induced by renal ischemia-reperfusion (I/R) injury. **a** In vivo detection of reactive oxygen species (ROS) in the rat kidneys subjected to sham-operation (Sham), ischemia-reperfusion surgery followed by intra-renal arterial injection (IA) of either phosphate-buffered saline (I/R+PBS), normoxia-cultured mesenchymal stem cells (I/R+MSC, 5 × 10^5^ cells per rat), or HMSCs (I/R+HMSC, 5 × 10^5^ cells per rat) was measured immediately after reperfusion. The amount of ROS was expressed as chemiluminescence counts/10 s. Please see the “Material and methods” for details. **b** Quantification of in vivo detection of the reactive oxygen species in the rat kidneys of Sham, I/R+PBS, I/R+MSC, and I/R+HMSC groups. **p < 0.01 by one-way ANOVA with Tukey’s post hoc comparison. ns, nonsignificant; *n* = 3 per group. **c** Representative periodic acid-Schiff stained images of kidney sections from rats subjected to sham operation (Sham group), renal ischemia for 45 min followed by 48 h of reperfusion and treated with either PBS, MSCs, or HMSCs at the onset of reperfusion. The I/R injury induced diffuse denudation of the renal tubular cells with loss of brush border (arrows) and flattening of tubular cells with intratubular cast formation (yellow arrowheads) in the I/R+PBS group. The extent of acute tubular injury (arrows) were decreased in the I/R+IA-MSC and I/R+IA-HMSC groups. Scale = 100 μm. **d** Quantification of the renal tubular injury. **p < 0.01, ***p < 0.001 by one-way ANOVA with Tukey’s post hoc comparison, *n* = 5 per group. **e**, **f** Serum blood urea nitrogen and creatinine levels in the Sham, I/R+PBS, I/R+IA-MSC, and I/R+IA-HMSC groups at 48 h after reperfusion. **p < 0.01, ***p < 0.001 by one-way ANOVA with Tukey’s post hoc comparison, *n* = 5 per group

Given that oxidative stress plays central roles in the progression of acute renal I/R injury [7, 44], next, we compare the anti-oxidative effects of HMSCs and normoxic MSCs. The rat model of renal I/R injury was established by clamping of the left renal artery for 45 min and right nephrectomy, followed by 48 h of reperfusion as previously described [34]. Either PBS, normoxic MSCs, or HMSCs were injected via the left renal artery immediately after the release of clamping. Because hypoxic MSCs would encounter a physiologically normoxic condition (21% O_2_) after intra-renal arterial (IA) or intraperitoneal (IP) administration into the rats with I/R injury, the beneficial gene expression while the HMSCs were transferred from the hypoxic to normoxic condition was first analyzed. We found that the hypoxia-derived upregulation of beneficial genes in HMSCs such as heparin-binding epidermal growth factor-like growth factor (*Hbegf*) can last for 48 h after the HMSCs were transferred to a normoxic atmosphere (Supplemental Figure S1). To further confirm that the HMSCs actually retained in the I/R-injured kidneys, the distribution of PKH67-labeled HMSCs was tracked after IA or IP administration. The green PKH67-labeled HMSCs can be identified in the renal tubules 48 h after the administration both in the IA and IP groups (Fig. 1c). The in vivo analyses of oxidative stress showed that I/R injury induced an abrupt increase of superoxide immediately after reperfusion, while administration of normoxic MSCs or HMSCs attenuated the increment of superoxide (Fig. 2a). Notably, HMSCs, but not normoxic MSCs, further suppressed the total oxidative content in the I/R-injured kidney homogenate as compared to PBS control (Fig. 2b). I/R injury resulted in severe renal tubular injury including diffuse denudation of tubular cells, tubular dilatation, and intratubular cast formation in the PBS group as compared to the sham-operated group. Intra-renal arterial administration of HMSCs decreased the I/R injury-induced renal tubular injury and the elevated blood urea nitrogen levels to a greater degree as compared to MSCs treatment (Fig. 2c–f). These results indicate that HMSCs have better anti-oxidative and renoprotective effects for renal I/R injury than normoxic MSCs.

### Effect of HMSCs by different administration routes on renal I/R injury

**Fig. 3:**
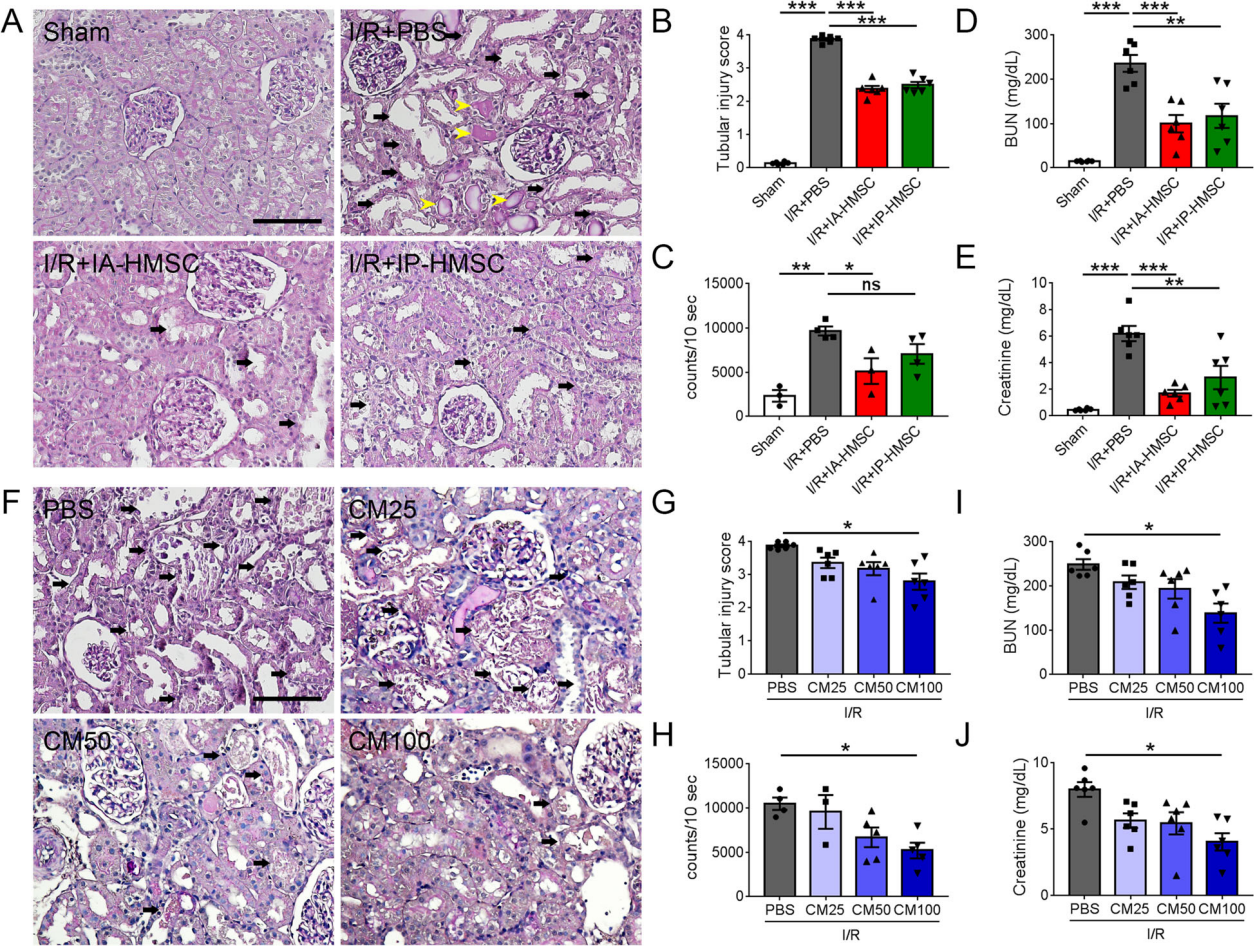
Hypoxic rat mesenchymal stem cells (HMSCs) and HMSC-conditioned medium (CM) suppressed oxidative stress, tubular injury, and renal dysfunction in ischemia-reperfusion (I/R)-injured rat kidneys. **a** Representative periodic acid-Schiff stained photomicrographs showed that I/R injury resulted in severe renal tubular injury such as tubular cell sloughing and flattening (arrows) as well as casts formation (yellow arrowheads) in phosphate-buffered saline (PBS)-treated groups as compared to the sham-operated (Sham) group. Scale = 100 μm. **b** Quantification of the tubular injury. Intra-renal arterial (IA-HMSC) or intraperitoneal (IP-HMSC) administration of HMSCs significantly reduced tubular injury. *n* = 6 per group. **c** Quantification of in vivo detection of the reactive oxygen species in the rat kidneys. I/R injury induced an increase of reactive oxygen species in the rat kidneys as compared to the Sham group. The I/R injury-induced oxidative stress was attenuated in the IA-HMSC group. *n* = 3–4 per group. **d**, **e** I/R injury caused a renal function decline with elevated serum blood urea nitrogen (BUN) and creatinine levels. Both intra-arterial and intraperitoneal administration of HMSCs improved the renal function decline significantly. *p < 0.05, **p < 0.01, ***p < 0.001 by one-way ANOVA with Tukey’s post hoc comparison. ns, nonsignificant; *n* = 6 per group. **f** Representative periodic acid-Schiff stained photomicrographs showed that the extent of sloughing and necrosis of the renal tubular cells due to I/R injury was gradually reduced by the HMSC-CM. CM25, CM50, and CM100 denote 25-fold, 50-fold, and 100-fold concentrated HMSC-CM, respectively. **g** Quantification of the tubular injury. *n* = 6 per group. **h** Quantification of in vivo detection of the reactive oxygen species in the rat kidneys. *n* = 3–5 per group. **i**, **j** Serum BUN and creatinine levels in the rats of renal I/R injury with treatment of PBS or HMSC-CM. Only 100-fold concentrated HMSC-CM (CM100) ameliorated oxidative stress, tubular injury and renal dysfunction. *p < 0.05 by one-way ANOVA with Tukey’s post hoc comparison, *n* = 6 per group

Substantially different outcomes have been observed when the same stem cell therapy is administered by different routes [21]. To further compare the efficacy between the local and systemic delivery modes, HMSCs were administered either intra-arterially through the left renal artery (IA) or via the intraperitoneal route (IP) immediately after I/R injury. I/R injury resulted in severe renal tubular injury including sloughing and flattening of tubular cells, tubular dilatation, and cast formation in the PBS group as compared to the sham-operated group. I/R injury also led to excessive ROS production and elevated serum BUN and creatinine levels in the PBS group. Both IA and IP administration of HMSCs limited the tubular injury and ameliorated renal function decline. Notably, only IA administration of HMSCs suppressed the I/R injury-induced excessive ROS as compared to the PBS group (Fig. 3a–e).

To further clarify whether the beneficial effects of HMSCs in renal I/R injury were through the direct homing or indirect paracrine effect, rats subjected to I/R injury were intraperitoneally injected with different concentration of HMSC-conditioned medium (HMSC-CM). There was a dose-response relationship among HMSC-CM treatment groups and 100-fold concentrated HMSC-CM significantly reduced renal I/R injury (Fig. 3f–j). These results suggest that HMSCs provide a renoprotective effect via suppressing excess oxidative stress in renal I/R injury.

### HMSCs enhanced anti-oxidant expression and inhibited superoxide generation in rats with I/R injury

**Fig. 4:**
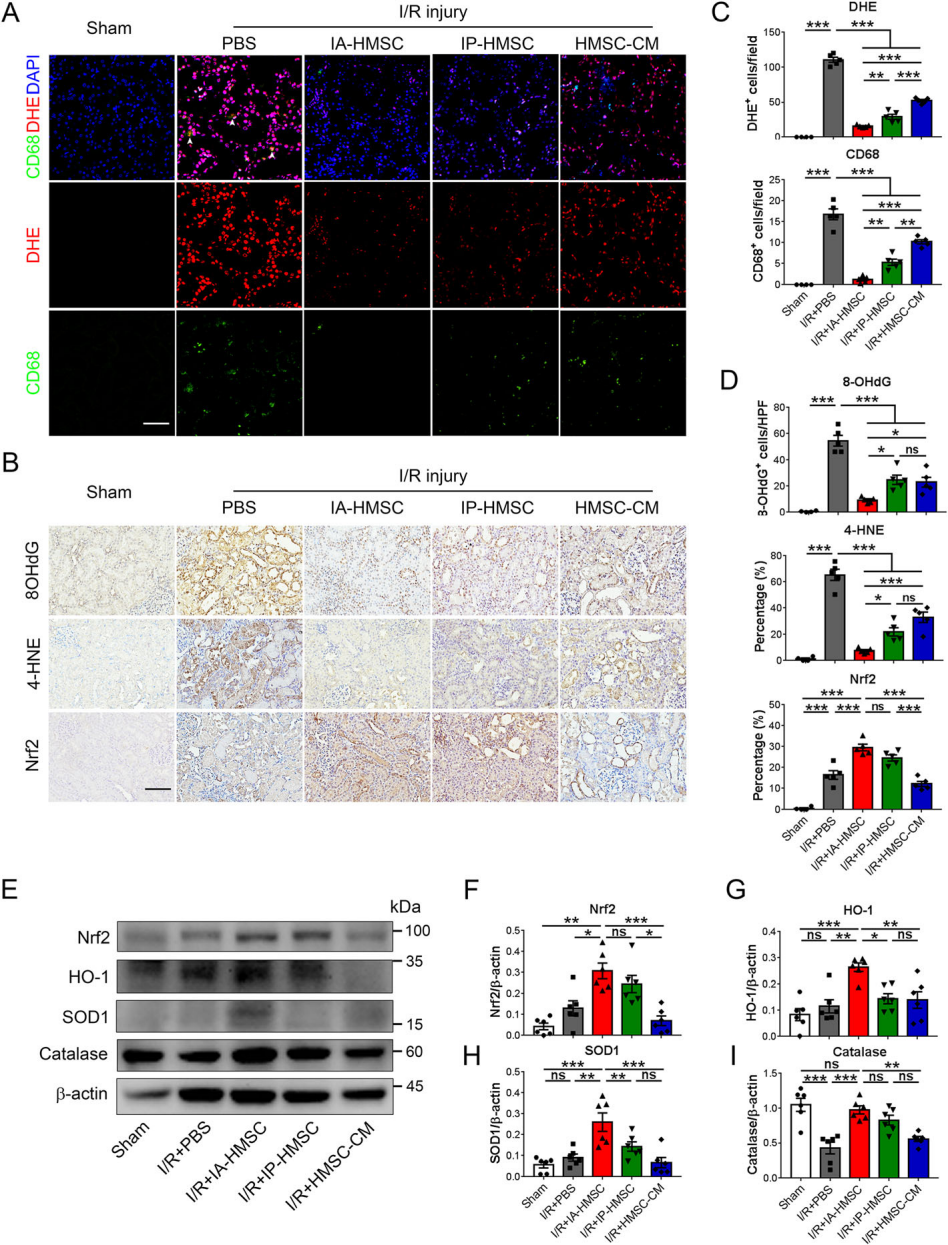
Hypoxic rat mesenchymal stem cells (HMSCs) decreased oxidative damage and increased anti-oxidant response in the ischemia-reperfusion (I/R)-injured rat kidneys. **a**, **c** Immunofluorescence staining for superoxide by dihydroethidium (DHE) staining and macrophage by anti-CD68 staining in renal tissues of the sham-operated rats (Sham), I/R-injured rats with PBS administration (PBS), I/R-injured rats with intra-renal arterial administration of HMSCs (IA-HMSCs), I/R-injured rats with intraperitoneal administration of HMSCs (IP-HMSCs), and I/R-injured rats with intraperitoneal administration of 100-fold concentrated HMSCs-conditioned medium (HMSC-CM). DHE staining was predominantly at the renal tubular cells and at some macrophages (arrowheads). 4′,6-Diamidino-2-phenylindole (DAPI) represented nuclear staining. Scale bar = 50 μm. **p < 0.01, ***p < 0.001 by one-way ANOVA with Tukey’s post hoc comparison, *n* = 4 for the sham group; *n* = 5 for other groups. **b**, **d** Immunohistochemical staining for 8-hydroxy-2-deoxyguanosine (8OHdG), 4-hydroxynonenal (4-HNE), and nuclear factor erythroid 2–related factor 2 (Nrf-2) in I/R-injured rat kidneys. Renal I/R injury increased the expression of 8OHdG, 4-HNE, and Nrf-2 in tubular epithelial cells. IA and IP administration of the HMSCs as well as intraperitoneal injection of HMSC-CM decreased the numbers of 8OHdG and 4-HNE-positive tubular cells but further upregulated the number of Nrf-2-positive tubular cells. Scale = 100 μm. *p < 0.05, **p < 0.01, ***p < 0.001 by one-way ANOVA with Tukey’s post hoc comparison. ns, nonsignificant. *n* = 4 for the sham group; *n* = 5 for other groups. **e**–**i** Western blot analyses of anti-oxidant proteins in I/R-injured rat kidneys. *p < 0.05, **p < 0.01, ***p < 0.001 by one-way ANOVA with Tukey’s post hoc comparison. ns, nonsignificant. *n* = 6 per group

Hypoxic culture during in vitro expansion has been shown to enhance the anti-oxidative properties of the MSCs [18]. Nonetheless, whether HMSCs ameliorate oxidative injury by increasing the endogenous anti-oxidant responses in renal I/R injury remains unclear. To further decipher the anti-oxidative effect of HMSCs, we then determined the production of superoxide in I/R-injured rats treated with HMSCs or HMSC-CM. DHE staining showed that the superoxide in the I/R-injured rat kidneys was predominantly generated from the renal tubular epithelial cells. Administration of HMSCs and HMSC-CM attenuated the formation of superoxide in the kidneys (Fig. 4a, c). We also determined the therapeutic potential of HMSCs and HMSC-CM on the DNA oxidation and lipid peroxidation products. I/R injury significantly increased the expression of 8-hydroxy-2-deoxyguanosine (8OHdG) and 4-hydroxynonenal (4-HNE) in renal tubular epithelial cells. Administration of HMSCs and HMSC-CM reduced these biomolecular damages induced by I/R injury (Fig. 4b, d). Additionally, I/R injury upregulated the expression of anti-oxidative nuclear factor erythroid 2–related factor 2 (Nrf-2) expression in I/R-injured rat kidneys as compared to the PBS group (Fig. 4b, d). Notably, IA administration of HMSCs not only further significantly enhanced the Nrf-2 expression but also increased heme oxygenase-1 (HO-1) and superoxide dismutase 1 (SOD1) expression (Fig. 4e–i). These data indicated that IA administration of HMSCs significantly suppressed superoxide formation, reduced DNA damage and lipid peroxidation, and enhanced anti-oxidants expression in renal tubular epithelial cells during I/R injury.

### HMSCs suppress inflammation and apoptosis in I/R-injured rat kidneys

**Fig. 5:**
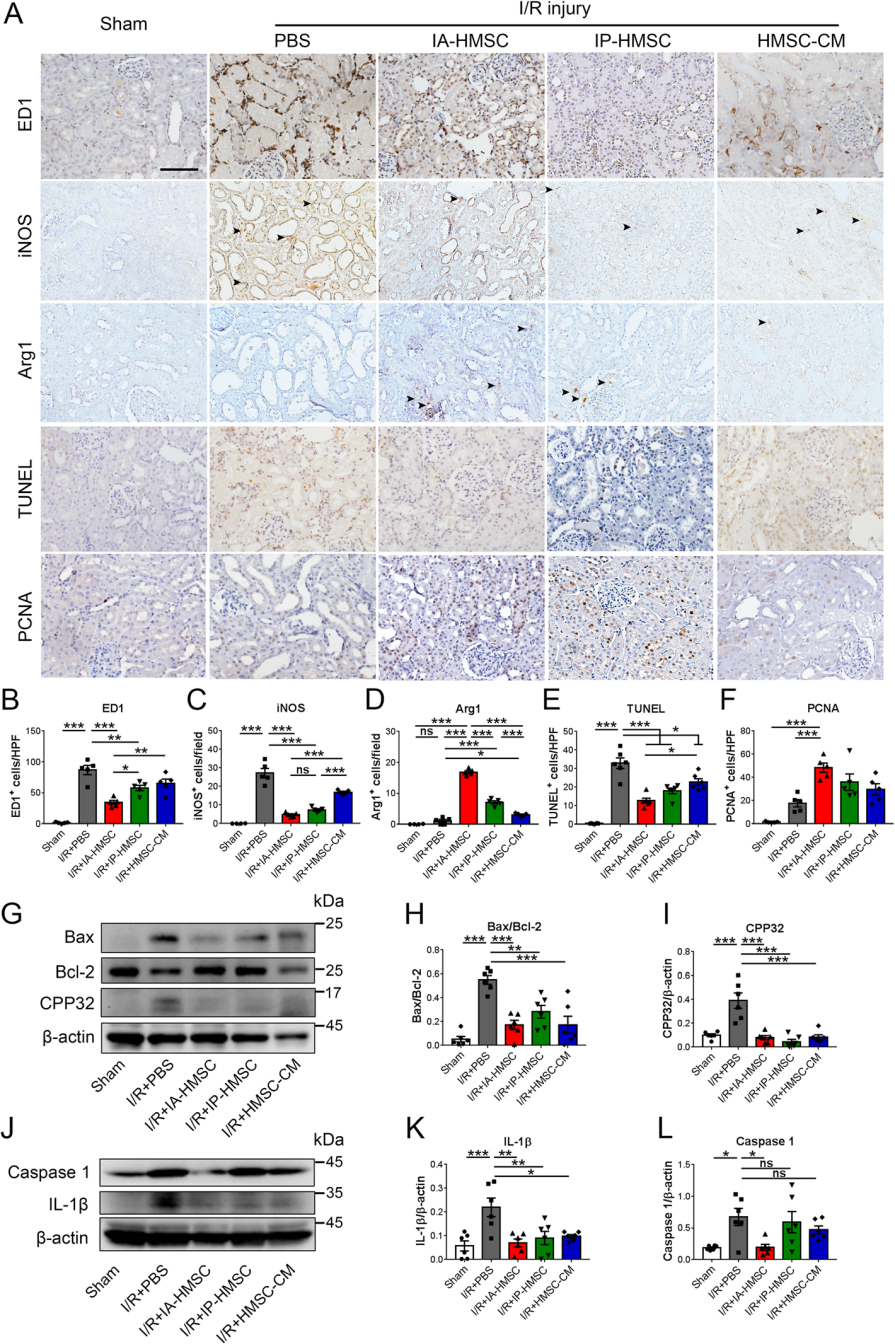
Hypoxic rat mesenchymal stem cells (HMSCs) modulated macrophage phenotypes, reduced tubular apoptosis, and enhanced tubular proliferation in the ischemia-reperfusion (I/R)-injured rat kidneys. **a**–**f** Representative immunohistochemical photomicrographs showed that the phosphate-buffered saline (PBS) group had more infiltrated ED1-positive and inducible nitric oxide synthase (iNOS)-positive macrophages, and terminal deoxynucleotidyl transferase dUTP nick end labeling (TUNEL)-positive apoptotic tubular cells as compared to the sham-operated (Sham) group. Intra-renal arterial (IA) and intraperitoneal (IP) administration of the HMSCs as well as intraperitoneal injection of the 100-fold concentrated HMSC-conditioned medium (CM) decreased the numbers of infiltrated iNOS-positive macrophages and TUNEL-positive apoptotic tubular cells. By contrast, renal I/R injury did not increase the number of arginase 1 (Arg1)-positive macrophages. IP administration of HMSCs as well as HMSC-CM significantly increased the number of Arg1-positive macrophages in I/R-injured rat kidneys. Only the IA-HMSC group had an increased number of proliferating cell nuclear antigen (PCNA)-positive tubular cells. Scale = 100 μm. *p < 0.05, **p < 0.01, ***p < 0.001 by one-way ANOVA with Tukey’s post hoc comparison, ns, nonsignificant, *n* = 4–5 for the sham group, *n* = 5–6 for other groups. **g**–**l** Western blot analyses showed that the I/R+PBS group had a higher ratio of Bax/Bcl-2 expressions and increased levels of active caspase 3 (CPP32), interleukin-1β (IL-1β) as well as caspase 1 in the I/R-injured rat kidneys. IA-HMSC, IP-HMSC, and HMSC-CM groups reduced the I/R-injury-induced elevation of Bax/Bcl2 ratio, CPP32, and IL-1β levels. Only IA-HMSC group supressed the I/R-injured-induced expression of caspase 1. *p < 0.05, ***p < 0.001 as compared to the sham-operated (sham) group; ^#^*p <* 0.05, ^##^*p <* 0.01, ^###^*p <* 0.001 as compared to the I/R+PBS group, by one-way ANOVA with Tukey’s post hoc comparison, *n* = 6 per group

The damaged renal tubular epithelial cells are not merely passive victims of I/R injury but also active attackers that generate chemotactic cytokines to attract inflammatory cells and perpetuate renal injury [5]. Macrophages are recruited early in the renal I/R injury and contribute to the ensuing interstitial inflammation and apoptosis of tubular cells [45]. Moreover, different macrophage subpopulations are recently found to direct the extent of tubular injury and repair [6]. Therefore, we next determined the effects of HMSCs and HMSC-CM treatment on the expression of inflammation markers and apoptosis in I/R-injured rat kidneys. I/R injury significantly increased the number of ED1-positive macrophages in the renal interstitium. Meanwhile, I/R injury also increased TUNEL-positive apoptotic renal tubular cells. Both IA and IP injection of HMSCs markedly suppressed ED1-positive macrophages and apoptotic tubular cells in I/R-injured kidneys. The reduction of macrophage infiltration and tubular apoptosis was lower in the IA group and that in the IP group. Moreover, I/R injury increased the interstitial infiltration of inducible nitric oxide synthase-positive M1 macrophage but not arginase 1-positive M2 macrophages. Administration of HMSCs or HMSC-CM significantly downregulated the M1 macrophage infiltration but upregulated M2 macrophages infiltration. Notably, HMSCs also augmented the expression of PCNA-positive renal tubular cells in rat I/R-injured kidney tissue (Fig. 5a–f). Moreover, the levels of apoptosis-related proteins in the I/R-injured kidneys were also examined. I/R injury increased the expression of activated Bax, caspase 3, caspase 1, and interleukin-1β but decreased the expression of Bcl-2. Both HMSCs and HMSC-CM treatment suppressed the expression of activated caspase 3 and interleukin-1β and decreased the Bax/Bcl-2 ratio in the I/R-injured kidneys. Nonetheless, only IA injection of HMSCs downregulated the expression of caspase 1 (Fig. 5g–l). These findings indicate that IA injection of the HMSCs has more pronounced anti-inflammatory, anti-apoptotic, and pro-regenerative effects in renal I/R injury.

### HMSCs enhance autophagy expression in I/R-injured rat kidneys

**Fig. 6:**
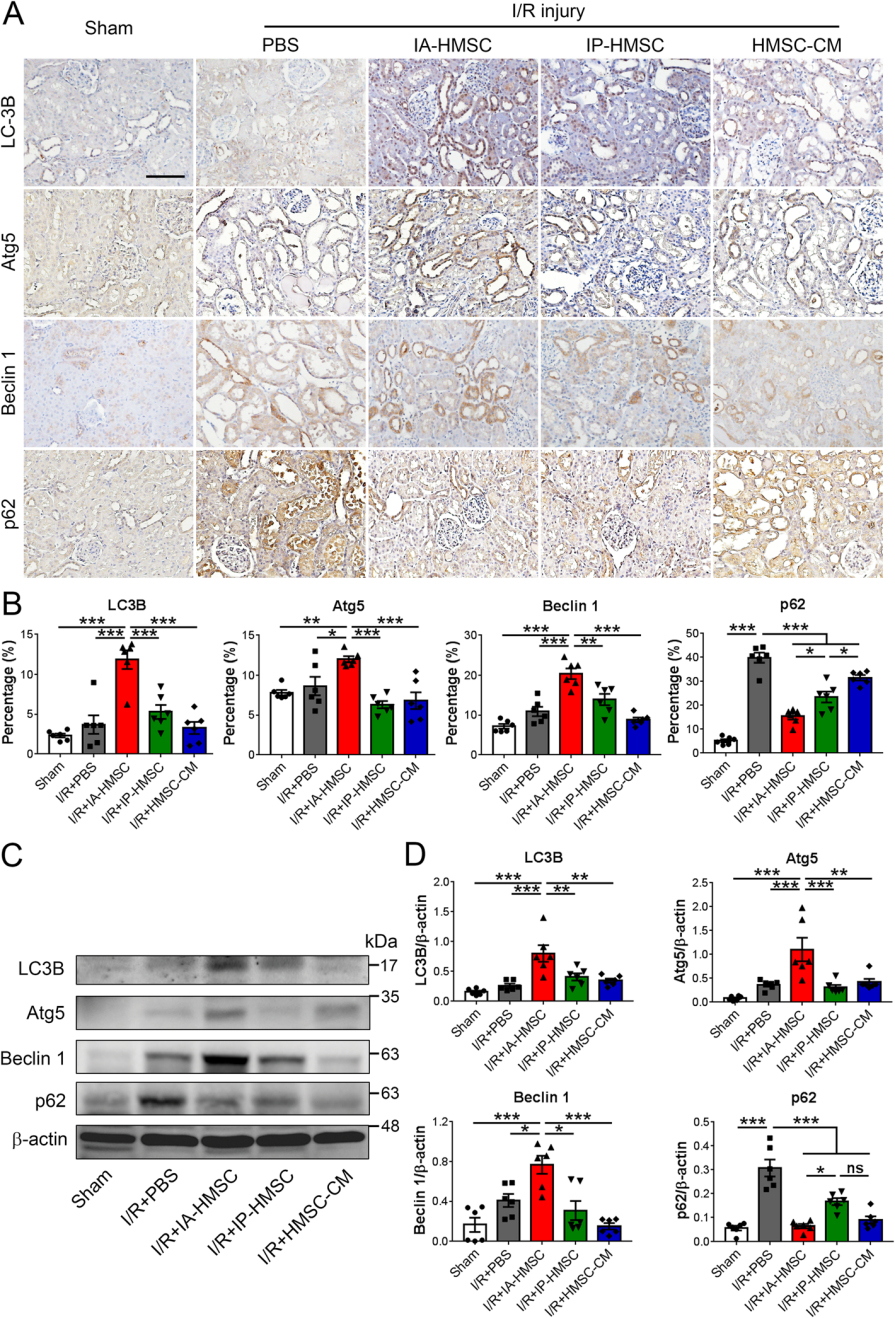
Hypoxic rat mesenchymal stem cells (HMSCs) upregulated autophagy in the tubular epithelial cells of the ischemia-reperfusion (I/R)-injured rat kidneys. **a**, **b** Representative immunohistochemical photomicrographs showed that intra-renal arterial (IA) administration of HMSCs upregulated autophagy-related protein expression including LC3B, Atg5, and Beclin 1 but downregulated the autophagy adaptor protein p62 expression in the renal tubular epithelial cells of I/R-injured rat kidneys. IP, intraperitoneal administration; CM, 100-fold concentrated conditioned medium. Scale = 100 μm. *p < 0.05, **p < 0.01, ***p < 0.001 by one-way ANOVA with Tukey’s post hoc comparison, *n* = 6 per group. **c**, **d** Western blot analyses showed that the I/R injury markedly increased the autophagy adaptor protein p62 expression, which was suppressed in the I/R+IA-HMSC, I/R+IP-HMSC, and I/R+HMSC-CM groups. The levels of LC3B, Atg5, and Beclin 1 were mildly increased in the I/R+PBS group but markedly increased in the I/R+HMSC group. *p < 0.05, **p < 0.01, ***p < 0.001 by one-way ANOVA with Tukey’s post hoc comparison. ns, nonsignificant. *n* = 6 per group

Mounting evidence indicates that autophagy is essential to maintain the function of renal proximal tubules during ischemic injury [10]. To further investigate whether HMSCs ameliorate renal I/R injury by modulating autophagy, we then determined the expression of autophagy in rat kidneys with or without I/R injury. Immunohistochemistry showed that the expression levels of autophagy-related protein including LC3B, Atg5, and Beclin 1 were increased in the renal tubular cells of the I/R-injured rat kidneys by IA administration of HMSCs (Fig. 6a, b). The western blot analysis also confirmed that IA administration of HMSCs significantly increased the expression of LC3B, Atg5, and Beclin 1. Consistently, the level of autophagy adaptor protein p62, which inversely correlates with autophagy activity, was also downregulated in the I/R-injured rat kidneys by IA injection of HMSCs (Fig. 6c, d). These results indicated that HMSCs enhance renal tubular autophagy in rats with renal I/R injury.

### HMSCs ameliorate H/R-injured renal tubular cells through upregulating autophagy

**Fig. 7:**
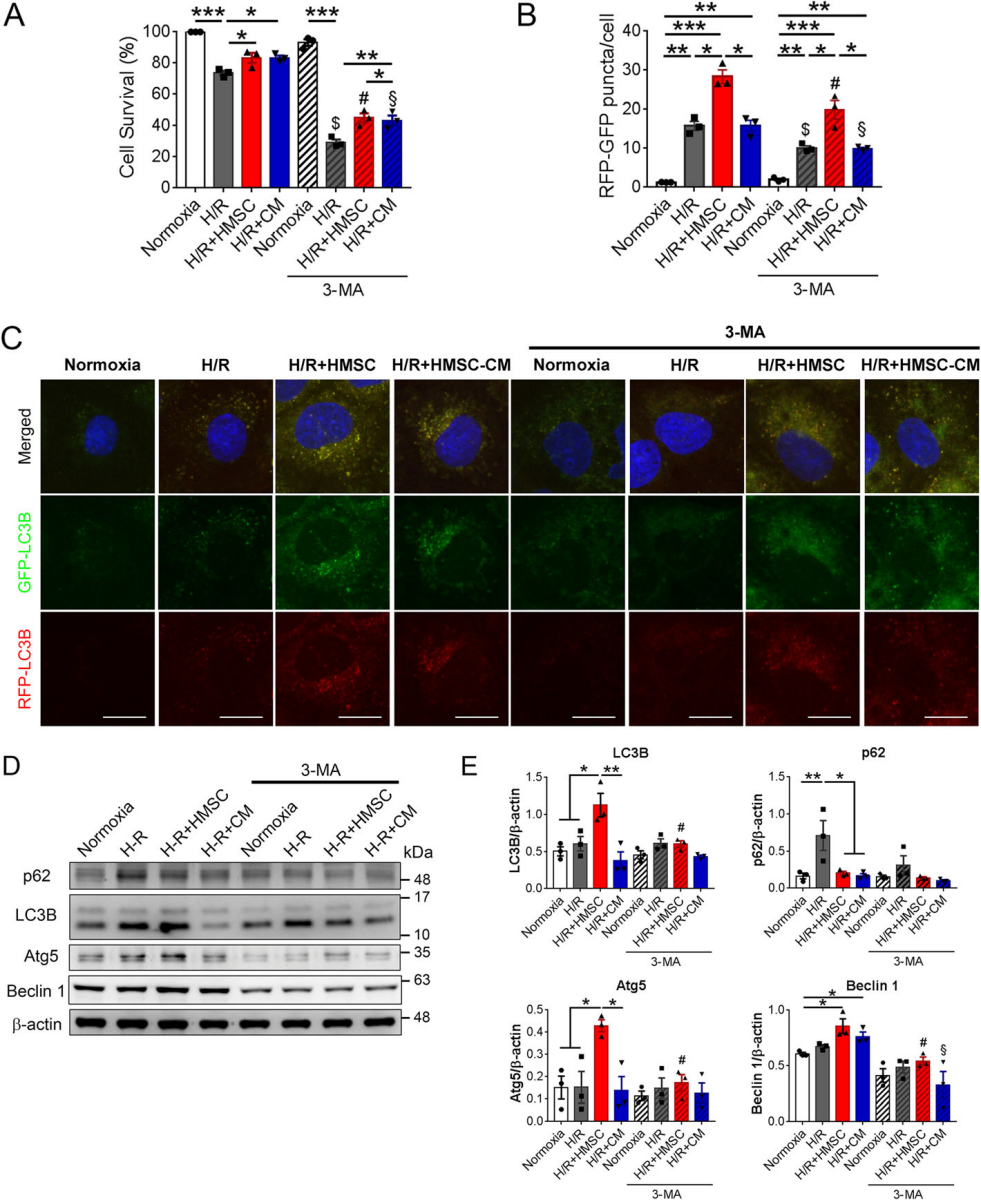
Hypoxic rat mesenchymal stem cells (HMSCs) ameliorated hypoxia-reoxygenation (H/R) injured renal tubular cells through enhancing autophagy. **a** H/R injury (hypoxia for 24 h followed by reoxygenation for 6 h) significantly decreased cell survival in NRK-52E cells determined by an MTT assay. Either co-culture with HMSCs or addition of HMSC-conditioned medium (CM) decreased H/R-injury-induced cell death. Addition of 3-methyl adenine (3-MA), an autophagy inhibitor, abolished the protective effects of HSMCs and HMSC-CM. *p < 0.05, **p < 0.01, ***p < 0.001 by one-way ANOVA with Tukey’s post hoc comparison. ^$^*p* < 0.05 vs H/R group; ^#^*p* < 0.05 vs H/R+HMSC group; ^§^*p* < 0.05 vs H/R+CM group. *n* = 3 per group. **b**, **c** Autophagic flux was assayed by a Premo Autophagy Tandem Sensor RFP-GFP-LC3B kit. Scale = 10 μm. Confocal microscopy showed that the LC3B puncta (yellow color) in the NRK-52E were increased by the co-culture with HMSCs or treatment with HMSC-CM. Addition of 3-MA decreased the formation of LC3B puncta. *p < 0.05, **p < 0.01, ***p < 0.001 by one-way ANOVA with Tukey’s post hoc comparison. ^$^*p* < 0.05 vs H/R group; ^#^*p* < 0.05 vs H/R+HMSC group; ^§^*p* < 0.05 vs H/R+CM group. *n* = 3 per group. **d**, **e** Western blot analyses showed that the H/R injury markedly increased the autophagy adaptor protein p62 expression in NRK-52E cells, which was suppressed in the H/R+HMSC and H/R+CM groups. The levels of LC3B, Atg5, and Beclin 1 were significantly increased in the H/R+HMSC group. Addition of 3-MA suppressed the HMSC-upregulated expression of LC3B, Atg5, and Beclin 1. *p < 0.05, **p < 0.01, ***p < 0.001 by one-way ANOVA with Tukey’s post hoc comparison. #*p* < 0.05 vs H/R+HMSC group by Student’s *t* test. *n* = 3 per group

To further confirm that HMSCs improve renal I/R injury via upregulating autophagy, we conducted an in vitro H/R injury model to simulate rats with I/R injury as previously described [34]. After being subjected to hypoxia for 24 h with subsequent reoxygenation for 6 h, rat renal proximal tubular NRK52E cells exhibited a significant lowering survival rate as compared to the normoxia-cultured group. Interestingly, co-culture with HMSCs or treatment with HMSC-CM both attenuated H/R injury-related death of NRK-52E cells. Nonetheless, addition of 3-methyladenine, an autophagy inhibitor, significantly abolished the beneficial effect of HMSCs and HMSC-CM (Fig. 7a). RFP-GFP-LC3B analysis of the autophagic flux showed that H/R injury induced the autophagy of NRK-52E cells and co-culture of HMSCs further promoted the autophagy flux. The expression of RFP-GFP-LC3B puncta was the most prominent in the HMSC co-culture group. However, addition of 3-MA decreased the expression of RFP-GFP-LC3B puncta (Fig. 7b–c). Western blotting also showed that co-culture with HMSCs upregulated the expression of LC3B, Atg5, and Beclin 1, and downregulated p62 expression in H/R-injured NRK-52E cells. Moreover, co-culture of HMSCs also increased the expression of anti-oxidants including Nrf-2, HO-1, SOD1, and catalase in H/R-injured NRK-52E cells (Supplemental Figure S2). Addition of 3-MA decreased the expression of autophagy proteins (Fig. 7d–e). Taken together, these data corroborated that HMSCs attenuate renal I/R injury through upregulating tubular autophagy in vitro.

### Increased autophagy level correlates with higher renal recovery in patients with AKI

**Fig. 8:**
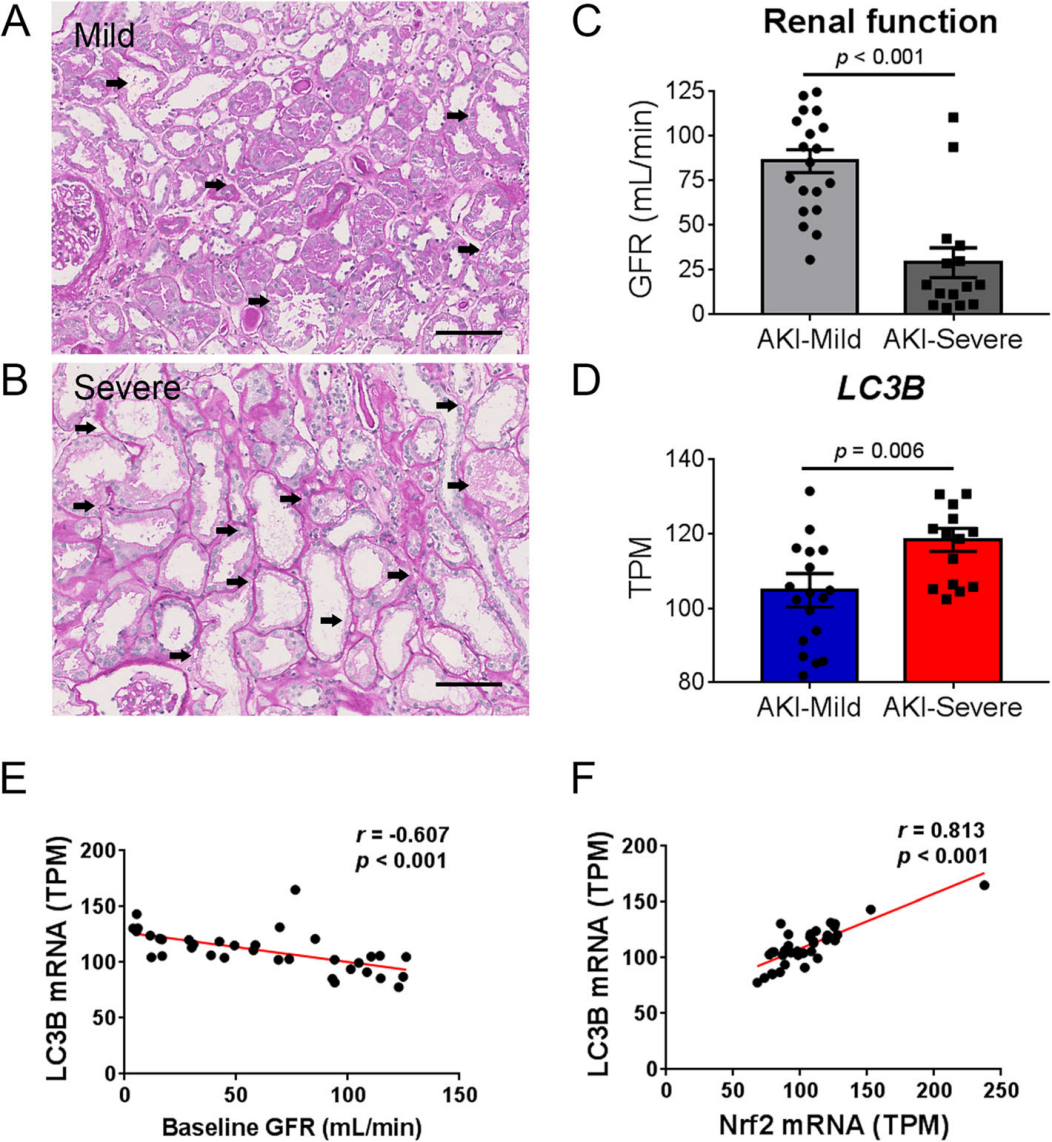
Tubular autophagy is associated with better renal recovery in patients with ischemic acute kidney injury (AKI). **a**, **b** Representative periodic acid-Schiff stained photomicrographs indicated the extent of acute tubular injury including loss of brush border, dilation of tubular lumens, and flattening or vacuolization of epithelial cells (arrows) in patients with mild (injured tubules < 50%) or severe (injured tubules ≥ 50%) ischemic AKI. Scale = 100 μm. **c** Baseline renal function in patients with mild (*n* = 20) and severe (*n* = 15, injured tubules ≥ 50%) ischemic AKI, compared by the unpaired *t* test. **d** Expression of LC3B mRNA levels (transcripts per million, TPM) in patients with mild and severe AKI, compared by the Wilcoxon rank sum test. **e**, **f** Correlation between renal LC3B expression with baseline renal function and Nrf2 in AKI patients by Pearson’s correlation test

To validate the beneficial effect of autophagy in renal I/R injury, we explore the role of LC3B expression in patients with ischemic AKI. The mRNA expression of LC3B was determined in the microdissected renal tubules of kidney biopsy specimens from patients with ischemic AKI. Patients with categorized into mild (injured tubules < 50%, n = 20) and severe (injured tubules ≥ 50%, n = 15) ischemic AKI groups according to the extent of tubular injury in the histological examination (Fig. 8a–b). We found that the severe AKI group had significant lower baseline glomerular filtration rate but higher extent of interstitial fibrosis and inflammation (Fig. 8c and Table 1). Interestingly, the LC3B expression was higher in the severe AKI group as compared to the mild AKI group (Fig. 8d). The renal LC3B mRNA expression was inversely correlated with baseline renal function but positively correlated with Nrf2 mRNA expression (Fig. 8e–f). There was no significant correlation between LC3B with HO-1, SOD1, catalase, or CD68 expression (Supplemental Figure S3). To further evaluate the prognostic role of LC3B expression in patients with AKI. Notably, multivariable regression analyses showed that a higher LC3B level in the biopsied kidney was associated with a greater chance of renal recovery (odds ratio of 3.93 for every 10 TPM increase of LC3B mRNA, Table 2). These clinical data further verify our experimental results that increased autophagy protect against renal I/R injury.

**Table 1:** Baseline characteristics of patients with mild or severe ischemic acute kidney injury

Baseline characteristics*	Mild AKI^**#**^ (n = 20)	Severe AKI^**#**^ (n = 15)	***p*** value^†^
**Demographics**			
Age, years	54.0 (26.3)	66.7 (12.4)	0.013^c^
Male	12 (60.0)	11 (73.3)	0.64^a^
Smoking	3 (15.0)	3 (20.0)	1.00^b^
Diabetes	3 (15.0)	4 (26.7)	0.43^b^
Hypertension	11 (55.0)	9 (60.0)	1.00^b^
**Laboratory and histological data**			
Serum creatinine, mg/dL	0.90 (0.47)	3.56 (4.01)	< 0.001^c^
Glomerular filtration rate, mL/min	89.2 (43.7)	16.7 (25.7)	< 0.001^c^
Interstitial fibrosis, %	10 (10)	30 (10)	< 0.001^c^
Interstitial inflammation, %	10 (7.5)	30 (20)	< 0.001^c^
LC3B mRNA levels, TPM	104 (25.1)	120 (19.9)	0.006^c^

*Values for categorical and continuous variables are expressed as numbers (percentages) and median (interquartile range), respectively

^**#**^Patients were stratified into mild (injured tubules < 50%) or severe (injured tubules ≥ 50%) ischemic acute kidney injury according to the percentage of injured tubules in the pathology of renal biopsy specimens

^†^Between-group comparisons by the ^a^Pearson’s *χ*^2^ test, ^b^Fisher’s exact test, or ^c^Wilcoxon rank sum test where appropriate

**Table 2:** Renal LC3B expression and recovery of renal function in patients with ischemic acute kidney injury^a^

		Odds ratio (95% CI)^c^	***p*** value^b^
**Renal LC3B expression (per 10 TPM increase)**^b^	Model 1: unadjusted	2.88 (1.50~7.46)	0.007
	Model 2: adjusted for age and gender	4.40 (1.80~16.82)	0.007
	Model 3: adjusted for age, gender and baseline renal function	3.94 (1.50~17.37)	0.024
	Model 4: adjusted for age, gender, baseline renal function, interstitial fibrosis, and inflammation	3.93 (1.40~21.55)	0.041

^a^Recovery of renal function was defined as the occurrence of a glomerular filtration rate greater than or equal to the baseline value during the follow-up period

^b^The LC3B mRNA levels in the renal biopsy specimens were expressed by TPM

^c^Odds ratio and 95% confidence interval (CI) for recovery of renal function were calculated by multivariable-adjusted logistic regression models

## Discussion

While MSCs have been reported to attenuate acute renal I/R injury, the detailed therapeutic mechanisms of the MSCs in this major clinical disease remains incompletely understood. In this report, we have demonstrated that hypoxia-preconditioned HMSCs had a greater anti-oxidative effect than normoxia-cultured MSCs in rats with renal I/R injury. We further found that HMSCs decreased renal interstitial inflammation, facilitated an M1-to-M2 macrophage transition, promoted renal tubular cell survival, and attenuated renal impairment in renal I/R injury. Mechanistically, we have discovered that HMSCs upregulated the autophagy expression both in the rats with renal I/R injury and in the renal proximal tubular cells with H/R injury. Notably, inhibition of the autophagy abrogated the renoprotective effect from HMSCs. The protective effect of tubular autophagy was also validated in clinical AKI patients. To the best of our knowledge, this is the first study to demonstrate that HMSCs attenuate renal I/R injury through enhancing tubular autophagy.

The most striking results of our study were that HMSCs attenuated renal I/R-related AKI through upregulating autophagy in renal tubular cells both in vivo and in vitro*.* Autophagy has been shown to exert protective roles in renal I/R injury [10, 11]. Nonetheless, current pharmacological inducers of autophagy like rapamycin and everolimus are limited to certain toxicities [10]. Although HMSCs play a renoprotective effect in AKI, it remains unclear that whether HMSCs lessen renal I/R injury through modulating the autophagy of renal tubular epithelial cells. We found that HMSCs upregulated the autophagy-related protein expression both in I/R-injured rat kidneys and H/R-injured renal tubular epithelial cells. After addition of 3-MA, the autophagy of renal tubular cells was inhibited and protective effect of HMSCs was then abrogated. Our data indicated that HMSCs attenuated I/R-induced renal tubular death through enhancing the autophagy in renal tubular cells. In accordance with our findings, Shin et al. recently found that administration of MSCs increase autophagosome and beclin-1 expression in the hippocampus of β-amyloid-treated mice and in the β-amyloid-treated neuronal cells, thereby promoting β-amyloid clearance and decreasing neuronal cell death [31]. Li et al*.* also showed that MSCs exert an autophagy-inducing effect in co-cultured pulmonary endothelial cells and attenuate acute lung injury [32]. Taken together, our novel finding that upregulation of renal tubular autophagy by HMSCs to repair renal I/R injury increases the understanding of the complex therapeutic mechanisms of HMSCs in AKI. Our study not only makes a conceptual breakthrough for understanding how HMSCs modulate renal tubular autophagy in I/R injury but also provides bench evidence to apply HMSCs to treat patients with ischemic AKI.

Oxidative stress is one of the major pathogenic mechanism of the I/R-related AKI [46–48]. Following renal ischemia, shortage of the oxygen and nutrients supply leads to tubular epithelial cell injury [5]. The injured epithelial cells and subsequent recruited pro-inflammatory macrophages produce large amount of ROS [47]. These highly reactive oxidizing molecules further contribute to tubular cell death through scavenging vasodilatory nitric oxide, destabilizing cytoskeleton, and increasing DNA oxidation and lipid peroxidation of plasma membrane [47]. ROS also perpetuate renal inflammation and trigger the expression of pro-inflammatory cytokines and chemokines [48]. Conversely, endogenous defense system like Nrf2, the downstream detoxifying enzymes, and other stress response proteins are activated during renal I/R injury to maintain cellular redox homeostasis [7, 49]. Increasing evidence suggests that oxidative stress and autophagy are intricately connected [48]. Mild oxidative stress induces cell repairing mechanisms such as autophagy [48]. Nonetheless, excessive production of ROS overwhelms the protective autophagy mechanism and leads to oxidative damage and ultimately cell demise [48]. Till now, the inter-connection between oxidative stress and autophagic processes in kidney diseases has not been investigated in detail. Our study revealed that I/R injury in rat kidneys induces a surge of ROS. DHE staining for characterization of in situ ROS further showed that the superoxide was predominantly from renal tubular epithelial cells in the renal I/R injury. The biomolecular damages from oxidative stress like 8OHdG and 4HNE were also increased in the I/R-injured renal tubular epithelial cells. Administration of HMSCs and HMSC-CM reduced the superoxide production, DNA oxidation, and lipid peroxidation in renal tubular cells of I/R-injured rat kidneys. Moreover, we also found that HMSC administration upregulated stress response proteins and detoxifying enzymes such as Nrf2, HO-1, SOD1, and catalase. Meanwhile, HMSC administration also enhances tubular autophagy, decreases tubular cell death, and attenuates renal function impairment. The autophagy adaptor p62 is recently found to interact with Keap1, the Nrf2 inhibitor, thus stabilizing and increased transcriptional activity of Nrf2 to prevent tissue damage and eventually to repair lesion and avoid cancerous lesion formation [50]. In accord with this, we found that HMSCs treatment simultaneously upregulated the autophagy pathway and Nrf2 expression in tubular epithelial cells of I/R-injured rat kidneys, suggesting these two systems may synergistically ameliorate renal injury. Clearly, the detailed interaction between autophagy and Nrf2 signaling promoted by HMSCs needs further investigation. Collectively, HMSCs treatment limits the extent of oxidative stress and activates tubular autophagy, both of which contributing to the protection of kidneys.

MSCs represent an appealing therapeutic approach for kidney diseases and their therapeutic efficacy is determined by the differentiation, trophic and immunomodulatory properties [51]. One critical aspect for the efficacy of MSCs is the delivery method and the optimal cell delivery technique would provide the greatest regenerative benefits and the least side effects [52]. In systemic administration through intravenous or intraperitoneal injection, most of the stem cells are trapped in the lung, liver, or spleen [53], and only few cells reach the injured site through homing effect [54, 55]. By contrast, direct intra-arterial injection of MSCs into the injured kidney would ensure the delivery. Our study also compared effect of locally delivery by intra-arterial route and systemic delivery by intraperitoneal route. We found that administration of HMSCs through intra-arterial route had better anti-oxidative and renoprotective effects in rats with I/R injury. By contrast, Moustafa et al*.* recently found that there was no difference between intra-arterial, intravenous or subcapsular injection of 5 × 10^6^ MSCs in cisplatin-induced AKI [56]. The discrepancy between Moustafa’s study and our report may be explained by the different dosage of stem cells and methods to induce AKI. Our previous study discloses that the best renoprotective effect of stem cells in renal I/R injury is achieved by administering 5 × 10^6^ stem cells because too many cells delivered intra-arterially also impede the renal perfusion [35]. Moreover, the present study also determined the effect of HMSC-CM to investigate the paracrine effect. Interestingly, the beneficial effect of HMSC-CM in renal I/R injury was only observed in the 100-fold concentrated group. Furthermore, the anti-inflammatory, anti-apoptotic, and regenerative effects of the HMSC-CM group were less prominent as compared to the IA administration of HMSC group. In line with our findings, a recent pilot clinical trial shows that intra-arterial infusion of autologous MSCs in patients with renovascular disease improves renal tissue oxygenation and cortical blood flow [57]. Therefore, the present study indicates that intra-arterial administration of HMSCs in I/R-related AKI is an effective and feasible delivery method.

Replicative exhaustion, early senescence, decreased differentiation potential, and impaired immunosuppressive ability remain major challenges of MSC therapy in clinical application [17]. Expansion of MSCs in the hypoxic atmosphere not only inhibits senescence, increases the proliferation rate, and improves differentiation ability but also enhances the paracrine effects through upregulation of various secretory trophic factors [26, 27]. Growing evidence suggests that hypoxia-preconditioning potentiates the therapeutic effects of MSC in renal I/R injury by enhancing the secretion of trophic factors like vascular endothelial growth factor, insulin-like growth factor-1, and hepatocyte growth factor [58]. The present study found that IA administration of HMSCs ameliorated I/R injury-induced renal impairment as compared that normoxic MSCs. We also demonstrated that the hypoxic culture increased the gene expression of hepatocyte growth factor and *Hbegf* as compared to normoxic culture. Notably, the hypoxia-derived upregulation of beneficial genes in HMSCs such as *Hbegf* lasted for 48 h after the HMSCs were transferred to a normoxic atmosphere. HBEGF has been implicated in angiogenesis [59] and enhances the proliferation of MSCs [60]. Exogenous HBEGF also accelerates renal recovery from acute ischemic injury by activating the epidermal growth factor receptor [61]. Altogether, our data indicate the hypoxia-derived upregulation of beneficial genes in MSCs can be maintained even after the cells were transferred from the hypoxic to normoxic condition to confer therapeutic effects.

Currently, MSCs-based therapy has been investigated in many clinical untreatable diseases [62]. While MSCs therapy is generally considered safe, there is still a concern regarding the tumorigenic risk after stem cell transplantation [62]. MSCs may promote tumor growth in the animal models of various solid malignancies by the secretion of proangiogenic factors [62]. By contrast, several studies have shown that MSCs can inhibit tumor progression and metastasis by inhibiting angiogenesis, downregulating Akt and Wnt signaling, and inducing apoptosis or cell cycle arrest [63]. A recent meta-analysis including 36 studies indicates that there is no association between MSCs therapy and tumor formation [64]. Administration of MSCs to patients of kidney diseases has also exhibited good safety and tolerability without tumor formation in clinical trials [17]. Moreover, there is also a dispute about the effect of hypoxia-preconditioning on the genetic stability of MSCs. A previous report demonstrated that low oxygen levels downregulated the expression of DNA repair proteins of MSCs [65]. However, latter studies have shown that a culture of MSCs at low oxygen tension improves genetic stability [66], and a long-term hypoxic culture may be more suitable for bone marrow-derived MSCs [67]. Till now, there is no unambiguous answer regarding the potential of MSCs in tumorigenesis. Use of MSCs expressing normal karyotype and genetic integrity help prevent tumorigenic transformation [27]. Long-term follow-up studies are still warranted to fully address the safety issue of MSCs-based therapy.

## Conclusions

In summary, our findings demonstrate that HMSCs exert anti-oxidative, anti-inflammatory, and anti-apoptotic effect in I/R-related AKI. We further found that HMSCs upregulated renal tubular autophagy, thereby attenuating renal tubular death, suppressing interstitial inflammation, and decreasing renal function impairment. The present study highlights the importance of modulation of tubular autophagy in repairing I/R injury, provides further mechanistic support for the reparative effects of HMSCs and facilitates the utilization of HMSCs in treating patients with ischemic AKI.

## Supplementary Information

**Additional file 1: Supplemental Materials and Methods.** **Figure S1.** Upregulation of beneficial genes of mesenchymal stem cells (MSCs) by hypoxic culture. **Figure S2.** Hypoxic rat mesenchymal stem cells (HMSCs) increased antioxidant response protein in hypoxia-reoxygenation (H/R)-injured renal tubular epithelial cells. **Figure S3.** Correlation between renal mRNA levels of LC3B with endogenous antioxidants and macrophage in patients with ischemic acute kidney injury. **Supplementary Table S1.** Primary and secondary antibodies list. **Supplementary Table S2.** Primer sequences and probe numbers in qPCR experiments.

## Data Availability

The datasets used and/or analyzed during the current study are available from the corresponding author on reasonable request.

## Abbreviations

3-MA: 3-Methyladenine
4-HNE: 4-Hydroxynonenal
8OHdG: 8-Hydroxy-2-deoxyguanosine
AKI: Acute kidney injury
Atg: Autophagy-related gene
BUN: Blood urea nitrogen
CM: Conditioned medium
DAPI: 4′,6-Diamidino-2-phenylindole
DHE: Dihydroethidium
DMEM: Dulbecco’s modified Eagle’s medium
FBS: Fetal bovine serum
FITC: Fluorescein isothiocyanate
H/R: Hypoxia-reoxygenation
Hbegf: Heparin-binding epidermal growth factor-like growth factor
SC: Hypoxic mesenchymal stem cell
HMSC-CM: Hypoxic mesenchymal stem cell-conditioned medium
HO-1: Heme oxygenase-1
I/R: Ischemia-reperfusion
IA: Intra-renal arterial
IP: Intraperitoneal
MCLA: 2-Methyl-6-(p-methoxyphenyl)-3,7-dihydroimidazo [1,2-alpha]pyrazin-3-one
MEM: Minimal essential medium
MSC: Mesenchymal stem cell
MTT: 3-[4,5-Dimethylthiazol-2-yl]-2,5-diphenyltetrazolium bromide
Nrf2: Nuclear factor erythroid 2–related factor 2
PAS: Periodic acid-Schiff
PBS: Phosphate-buffered saline
ROS: Reactive oxygen species
SOD1: Superoxide dismutase 1
TUNEL: Terminal deoxynucleotidyl transferase-mediated digoxigenin-deoxyuridine triphosphate nick-end labeling
