# Supplementary material for: Hypoxic mesenchymal stem cells ameliorate acute kidney ischemia-reperfusion injury via enhancing renal tubular autophagy
Source: Stem Cell Res Ther. 2021 Jun 28;12:367. doi: 10.1186/s13287-021-02374-x (PMC8240301; doi:10.1186/s13287-021-02374-x)
Supplement: Supplementary file 1 — Additional file 1: Supplemental Materials and Methods. Figure S1. Upregulation of beneficial genes of mesenchymal stem cells (MSCs) by hypoxic culture. Figure S2. Hypoxic rat mesenchymal stem cells (HMSCs) increased antioxidant response protein in hypoxia-reoxygenation (H/R)-injured renal tubular epithelial cells. Figure S3. Correlation between renal mRNA levels of LC3B with endogenous antioxidants and macrophage in patients with ischemic acute kidney injury. Supplementary Table S1. Primary and secondary antibodies list. Supplementary Table S2. Primer sequences and probe numbers in qPCR experiments. [file 13287_2021_2374_MOESM1_ESM.docx]

**Supplementary Data**

**Hypoxic mesenchymal stem cells ameliorate acute kidney ischemia-reperfusion injury via enhancing renal tubular autophagy**

**Supplemental Materials and Methods**

***RNA extraction and real-time PCR***

Total RNA was extracted using the NuleoZOL (Macherey-Nagel, Düren, Germany) from the cells according to manufacturer’s instructions. The mRNA levels were measured by the quantitative real-time PCR using the Taqman hydrolysis probe system (Roche Applied Science) on a LightCycler 1.5 Instrument (Roche Applied Science) as previously described [1]. Primers and probes were designed by the Universal Probe Library Assay Design Center and verified by the NCBI BLAST. Data were normalized to *Actb* (β-actin encoding gene) expression. Nuclease-free water will be used as the negative control template. The primer sequences and probe numbers were listed in Supplemental Table S2.

**Supplemental Reference**

1. Tseng WC, Chuang CW, Yang MH, Pan CC, Tarng DC. Kruppel-like factor 4 is a novel prognostic predictor for urothelial carcinoma of bladder and it regulates TWIST1-mediated epithelial-mesenchymal transition. Urol Oncol. 2016;34:485 e15- e24.

**Figure S1. Upregulation of beneficial genes of mesenchymal stem cells (MSCs) by hypoxic culture.**

Quantitative polymerase chain reaction analyses of (A) hypoxia response gene and (B-D) genes encoding for beneficial growth factors in MSCs under normoxia (21% O_2_), MSCs under hypoxia (1% O_2_) and MSCs transferred from hypoxia to normoxia for 48 hours (Hypoxia 🡪 Normoxia). **p <*0.05, ***p <*0.01 by one-way ANOVA with Tukey’s *post hoc* comparison. ns, non-significant. n = 3 per group.


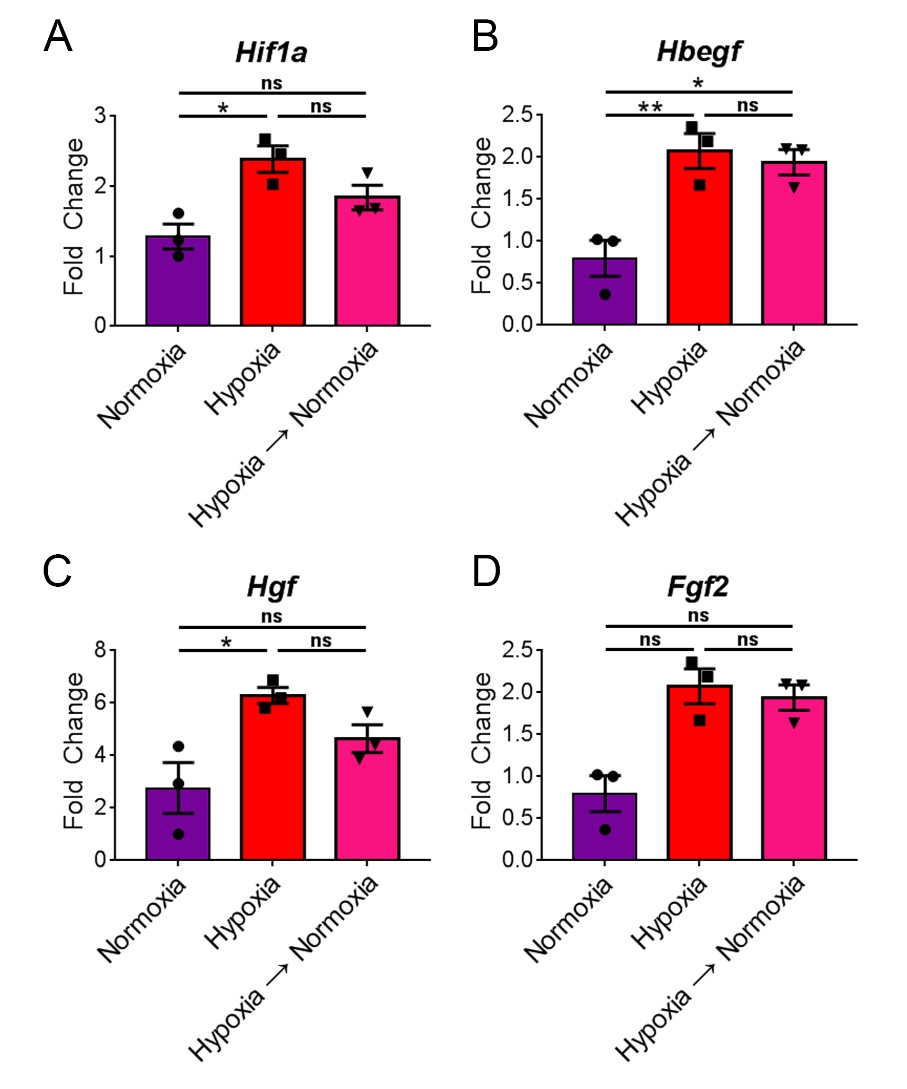


**Figure S2. Hypoxic rat mesenchymal stem cells (HMSCs) increased antioxidant response protein in hypoxia-reoxygenation (H/R)-injured renal tubular epithelial cells**

Western blot analyses showed that the H/R (hypoxia for 24 hours followed by reoxygenation for 6 hours) injury increased the expression levels of nuclear factor erythroid 2–related factor 2 (Nrf-2), heme oxygenase-1 (HO-1), superoxide dismutase 1 (SOD1) and catalase and in NRK-52E cells. Transwell coculture with HMSCs or addition of HMSC-conditioned medium (HMSC-CM) further increased the expression of these antioxidant protein.

**
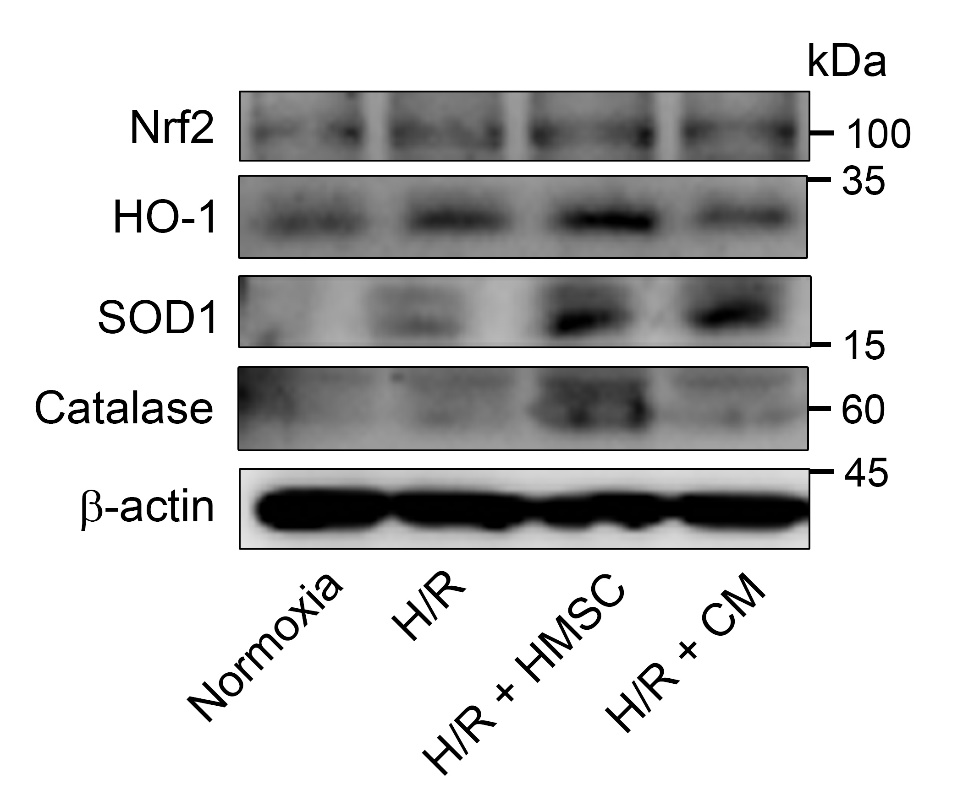
**

**Figure S3. Correlation between renal mRNA levels of LC3B with endogenous antioxidants and macrophage in patients with ischemic acute kidney injury.** LC3B versus (A) heme oxygenase 1 (HO-1), (B) superoxide dismutase 1 (SOD1), (C) catalase and (D) CD68 by Pearson’s correlation test.


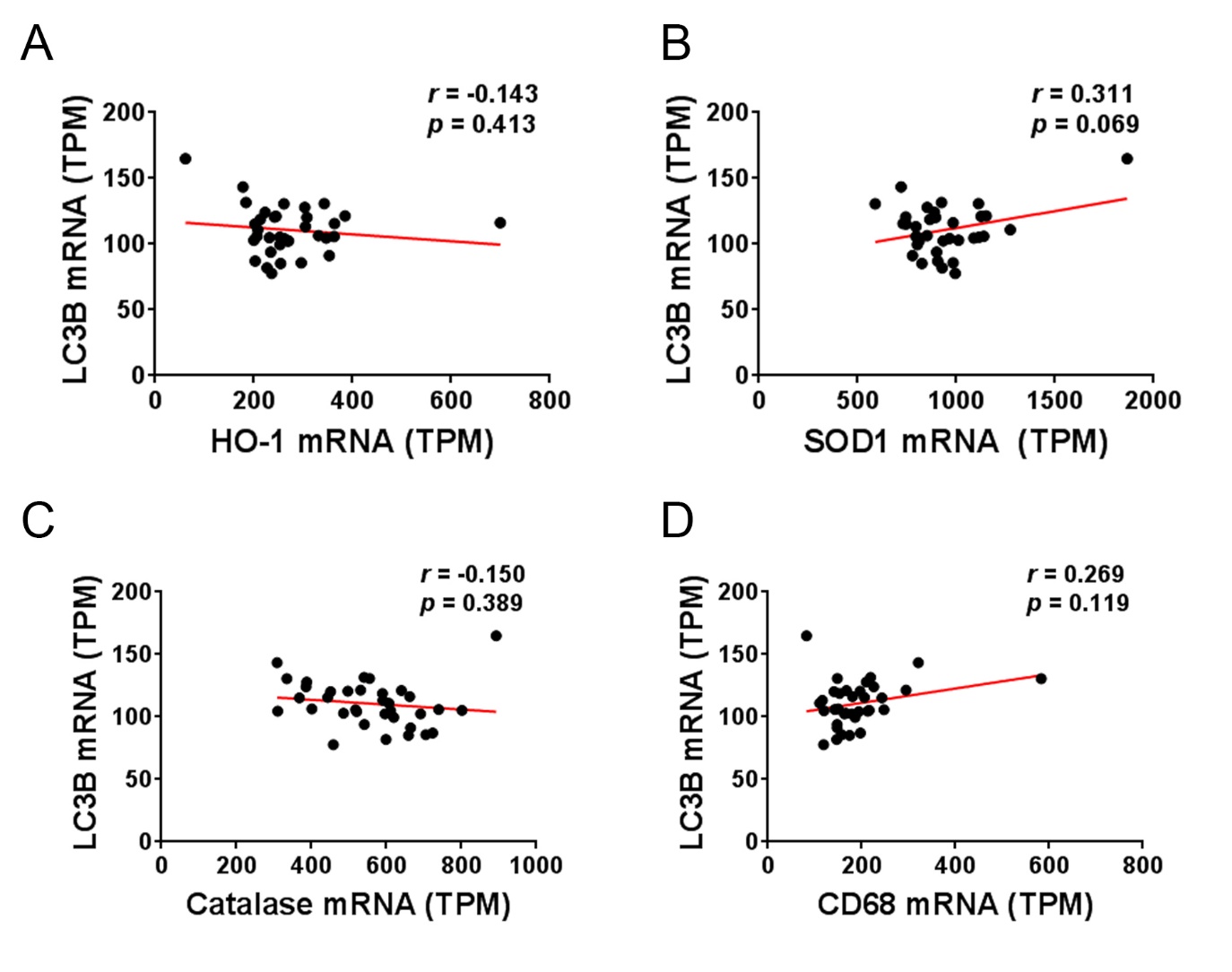


| **Supplementary Table S1. Primary and secondary antibodies list** | | | | |
| --- | --- | --- | --- | --- |
| **Reagent or Resource** | **Source** | **Dilution** | **Incubation condition** | **Cat#** |
| ***Western blotting*** |  |  |  |  |
| ***Primary antibodies*** |  |  |  |  |
| Bax | Cell Signaling | 1:1000 | 4^o^C O/N | 2857S |
| Bcl-2 | Cell Signaling | 1:1000 | 4^o^C O/N | 2870S |
| CPP32 | Sigma-Aldrich | 1:1000 | 4^o^C O/N | C8487 |
| Caspase-1 | Abcam | 1:1000 | 4^o^C O/N | ab108362 |
| IL-1β | Santa Cruz Biotechnology | 1:1000 | 4^o^C O/N | sc-7884 |
| LC-3B | ProteinTech | 1:1000 | 4^o^C O/N | 11306-1-AP |
| Atg5 | ProteinTech | 1:1000 | 4^o^C O/N | 10181-2-AP |
| Beclin 1 | ProteinTech | 1:1000 | 4^o^C O/N | 11306-1-AP |
| p62 | ProteinTech | 1:1000 | 4^o^C O/N | 18420-1-AP |
| Catalase | ProteinTech | 1:2000 | 4^o^C O/N | 21260-1-AP |
| HO-1 | ProteinTech | 1:2000 | 4^o^C O/N | 10701-1-AP |
| SOD1 | ProteinTech | 1:2000 | 4^o^C O/N | 10269-1-AP |
| Nrf2 | ProteinTech | 1:2000 | 4^o^C O/N | 16396-1-AP |
| β-actin | ProteinTech | 1:5000 | 4^o^C O/N | 60008-1-Ig |
| ***Secondary antibodies*** |  |  |  |  |
| Goat anti-rabbit IgG | Jackson ImmunoResearch | 1:10000 | RT 1.5 h | 111-035-003 |
| Goat anti-mouse IgG | AbRaY | 1:10000 | RT 1.5 h | RA-BZ102 |
| ***Immunofluorescence*** |  |  |  |  |
| Dihydroethidium | Sigma-Aldrich | 100 μM | RT 15 min | D7008 |
| CD68 | Genetex | 1:100 | 4^o^C O/N | GTX41868 |
| FITC | Jackson ImmunoResearch | 1:250 | RT 2 h | 115-095-003 |
| DAPI | Sigma-Aldrich | 1:250 | RT 20 min | D9542 |
|  |  |  |  |  |
| ***Immunohistochemistry*** |  |  |  |  |
| ED1 | AbD Serotec | 1:100 | 4^o^C O/N | MCA-341 |
| PCNA | Santa Cruz Biotechnology | 1:100 | 4^o^C O/N | sc-56 |
| LC-3B | ProteinTech | 1:100 | 4^o^C O/N | 11306-1-AP |
| Atg5 | ProteinTech | 1:200 | 4^o^C O/N | 10181-2-AP |
| Beclin 1 | ProteinTech | 1:200 | 4^o^C O/N | 11306-1-AP |
| p62 | ProteinTech | 1:100 | 4^o^C O/N | 18420-1-AP |
| 8-OHdG | Abcam | 1:200 | 4^o^C O/N | ab62623 |
| 4-HNE | Abcam | 1:100 | 4^o^C O/N | ab46545 |
| Nrf2 | ProteinTech | 1:200 | 4^o^C O/N | 16396-1-AP |
| iNOS | Santa Cruz Biotechnology | 1:200 | 4^o^C O/N | sc-651 |
| Arg1 | Santa Cruz Biotechnology | 1:200 | 4^o^C O/N | sc-20150 |
| ***Flow Cytometry*** |  |  |  |  |
| CD29 | Biolegend | 1:25 | 4^o^C 1h | 102205 |
| CD44 | Biolegend | 1:25 | 4^o^C 1h | 203906 |
| CD73 | Bioss | 1:15 | 4^o^C 1h | bs-4834R-FITC |
| CD90 | Biolegend | 1:25 | 4^o^C 1h | 206105 |
| CD105 | Bioss | 1:15 | 4^o^C 1h | bs-4609R-FITC |
| CD45 | eBioscience | 1:25 | 4^o^C 1h | 11-0461-80 |
| CD11b | Biolegend | 1:20 | 4^o^C 1h | 201807 |
| CD31 | Santa Cruz Biotechnology | 1:100 | 4^o^C 2h | sc-1506-R |
| FITC | Jackson ImmunoResearch | 1:100 | 4^o^C 1h | 111-095-003 |

| **Supplementary Table S2. Primer sequences and probe numbers in qPCR experiments** | | |
| --- | --- | --- |
| **Gene** | **Primer sequence** | **Probe number** |
| For Rat hypoxic and normoxic mesenchymal stem cells | | |
| *Hbegf* F | caggacttggaagggacaga | 25 |
| *Hbegf* R | tttcccattcctttctttgc |  |
| *Hgf* F | gattggatcaggaccttgtga | 49 |
| *Hgf* R | ccattctcattttgtgttgttca |  |
| *Fgf2* F | tcttcctgcgcatccatc | 7 |
| *Fgf2* R | gcttggagctgtagtttgacg |  |
| *Hif1a* F | tggaagcactagacaaagctca | 95 |
| *Hif1a* R | ttgaccatatcgctgtccac |  |
| *Actb* F | cccgcgagtacaaccttct | 17 |
| *Actb* R | cgtcatccatggcgaact |  |
| Abbreviation: Hbegf, Heparin binding EGF like growth factor; Hgf, Hepatocyte growth factor; Fgf2, Fibroblast growth factor 2; Hif1a, Hypoxia inducible factor 1 subunit alpha, Actb, Actin beta; F, Forward, R, Reverse. | | |
